# Genomic Analysis of Mouse Retinal Development

**DOI:** 10.1371/journal.pbio.0020247

**Published:** 2004-06-29

**Authors:** Seth Blackshaw, Sanjiv Harpavat, Jeff Trimarchi, Li Cai, Haiyan Huang, Winston P Kuo, Griffin Weber, Kyungjoon Lee, Rebecca E Fraioli, Seo-Hee Cho, Rachel Yung, Elizabeth Asch, Lucila Ohno-Machado, Wing H Wong, Constance L Cepko

**Affiliations:** **1**Department of Genetics and Howard Hughes Medical Institute, Harvard Medical SchoolBoston, Massachusetts, United States of America; **2**Dana-Farber Cancer Institute, Harvard Medical SchoolBoston, MassachusettsUnited States of America; **3**Department of Statistics, University of CaliforniaBerkeley, CaliforniaUnited States of America; **4**Children's Hospital Informatics Program, BostonMassachusettsUnited States of America; **5**Decision Systems Group, Brigham and Women's HospitalBoston, MassachusettsUnited States of America; **6**Department of Biostatistics, Harvard School of Public HealthBoston, MassachusettsUnited States of America

## Abstract

The vertebrate retina is comprised of seven major cell types that are generated in overlapping but well-defined intervals. To identify genes that might regulate retinal development, gene expression in the developing retina was profiled at multiple time points using serial analysis of gene expression (SAGE). The expression patterns of 1,051 genes that showed developmentally dynamic expression by SAGE were investigated using in situ hybridization. A molecular atlas of gene expression in the developing and mature retina was thereby constructed, along with a taxonomic classification of developmental gene expression patterns. Genes were identified that label both temporal and spatial subsets of mitotic progenitor cells. For each developing and mature major retinal cell type, genes selectively expressed in that cell type were identified. The gene expression profiles of retinal Müller glia and mitotic progenitor cells were found to be highly similar, suggesting that Müller glia might serve to produce multiple retinal cell types under the right conditions. In addition, multiple transcripts that were evolutionarily conserved that did not appear to encode open reading frames of more than 100 amino acids in length (“noncoding RNAs”) were found to be dynamically and specifically expressed in developing and mature retinal cell types. Finally, many photoreceptor-enriched genes that mapped to chromosomal intervals containing retinal disease genes were identified. These data serve as a starting point for functional investigations of the roles of these genes in retinal development and physiology.

## Abstract 2

Spatial and temporal patterns of expression for over 1000 genes in identified retinal cells invites functional investigations into the role of these genes in development and physiology

## Introduction

The vertebrate retina is a model system for studying both the development and function of the central nervous system (CNS). Only six major types of neurons develop within the retina, along with a single type of glial cell (Rodieck 1998). These cells are readily distinguished from one another by morphology and laminar position within the retina. Birthdating studies have shown that retinal cell types are generated in overlapping intervals, with ganglion cells, cone photoreceptors, amacrine cells, and horizontal cells generated prior to birth, and bipolar neurons and Müller glia generated after birth in mice (Sidman 1961; Young 1985a, 1985b). Rod photoreceptors, the most abundant retinal cell type in the retina, are born both pre- and postnatally, with a peak of genesis coincident with the day of birth in the mouse.

These birthdating studies, together with heterochronic coculture experiments (Belliveau and Cepko 1999; Belliveau et al. 2000; Rappaport et al. 2001), heterochronic transplantation (Rappaport et al. 2001), and lineage analysis (Turner and Cepko 1987; Holt et al. 1988; Wetts and Fraser 1988; Turner et al. 1990), have given rise to the competence model of retinal cell fate specification (Cepko et al. 1996). The competence model states that the intrinsic ability of mitotic retinal progenitor cells to produce a particular cell fate changes continually through development. A cell produces only a single fate, or a subset of fates, at any one time even though lineage analysis has shown that most retinal progenitors have the potential to produce many or all fates over the entire period of retinal development. Interestingly, even at one time in development, retinal progenitor cells show heterogeneity in their developmental competence (Alexiades and Cepko 1997; Belliveau and Cepko 1999; Belliveau et al. 2000; Rapaport et al. 2001). In addition to the contribution of intrinsic determinants of cell fate specification, the fates chosen by the daughters of a retinal progenitor may be influenced by extrinsic factors (Watanabe and Raff 1990; Altschuler et al. 1993; Kelley et al. 1994; Levine et al. 1997, 2000; Belliveau and Cepko 1999; Young and Cepko 2004). Finally, certain aspects of retinal cell fate choice, such as the specification of at least some rod and bipolar cells, appear to occur in postmitotic cells (Ezzeddine et al. 1997).

Although the competence model was formulated to explain cell fate choice in the retina, it is clear that cell specification in many other regions of the developing nervous system—including neural crest (Selleck and Bronner-Fraser 1996), spinal cord (Ericson et al. 1996), and cerebral cortex (McConnell 1988; Qian et al. 2000)—involve changes in progenitor competence over time, frequently resulting in altered sensitivity to extrinsic factors. The model of temporal changes in competence is strongly supported by recent elegant studies of *Drosophila* CNS development (Isshiki et al. 2001; Pearson and Doe 2003), where a temporal order of transcription factor expression was found to set the context of cell fate determination. The fundamental similarity among these systems nonetheless accommodates mechanistic differences. The situation in the retina, where early progenitor cells cannot be induced to adopt late fates and vice versa (although see James et al. [2003] for a possible exception to this rule), is distinct from the progressive developmental restriction that is seen in the cerebral cortex, where early cortical progenitor cells are competent to generate cells of upper (late-born) and lower (early-born) layers of the cortex, but become restricted to generating only late-born fates as development proceeds (Desai and McConnell 2000).

It is not known what genes mediate changes in progenitor competence during retinal development. Likewise, it is not known to what extent individual retinal progenitor cells from a single time point differ in their developmental competence from one another, although a few genes that are expressed in distinct subsets of progenitor cells have been found (Austin et al. 1995; Matter et al. 1995; Alexiades and Cepko 1997; Dyer and Cepko 2000a; Brown et al. 2001; Wang et al. 2001). Moreover, the genes that regulate the differentiation of any retinal cell type following commitment to a specific fate are generally poorly understood, although a number of transcription factors such as Crx, Nrl, and NR2E3 (Chen et al. 1997; Furukawa et al. 1997a; Haider et al. 2001; Mears et al. 2001) are clearly important in rod development. Unbiased, comprehensive expression profiling studies offer the possibility of identifying the molecular components and networks underlying these processes, as well as revealing target genes involved in intermediate and terminal differentiation of individual retinal cell types.

We have used serial analysis of gene expression (SAGE) to profile gene expression during the development of the mouse retina (Blackshaw et al. 2001). SAGE, which provides an unbiased and nearly comprehensive readout of gene expression, is conceptually very much like expressed sequence tag (EST) sequencing, with the difference being that concatenated libraries of short sequence tags derived from each cDNA found in the sample of interest are sequenced (Velculescu et al. 1995). By identifying genes that show dynamic expression via SAGE and testing the cellular expression of these genes via in situ hybridization (ISH), we can identify genes that potentially regulate proliferation, cell fate determination, and cell differentiation. Furthermore, by examining SAGE libraries made from adult tissue, genes that are specifically expressed in mature cell types can be identified.

By employing both SAGE-based expression profiling and large-scale ISH analysis to determine cellular expression of developmentally dynamic transcripts, we aim to combine the strengths of these two approaches and obtain a detailed picture of molecular events taking place during development of the retina. The laminar structure of the retina, which allows identification of the major cell types expressing a transcript under examination, makes large-scale ISH particularly informative relative to many other regions of the nervous system.

## Results/Discussion

### Summary of SAGE Data

SAGE was conducted on mouse retinal tissue taken at 2-d intervals from near the start of neurogenesis at embryonic day 12.5 (E12.5) to nearly the end of neurogenesis at postnatal day 6.5 (P6.5). In addition, libraries were made from P10 wild-type mice and the adult retina. Previously generated SAGE data from the microdissected outer nuclear layer (ONL) of the retina, which comprises roughly 97% rod photoreceptors, from retinal tissue from mice that were deficient for Crx (littermates of the wild-type P10 mice), and from adult hypothalamus were also incorporated into the analysis (Blackshaw et al. 2001). All of these libraries were sequenced to a depth of 50,000–60,000 SAGE tags each 14 bp long. Table S1 lists the number of distinct tags found in the 12 retina1 libraries and their abundance levels, along with the number of tags that do not match any known transcript. While 10% of all unique tags found twice or more in the 12 libraries did not correspond to an identified transcript, only 3% of the tags found five times or more did not match a known transcript (Table S1). Table S2 lists all individual tag levels in each of these retinal libraries, along with data from a number of other publicly available nonretinal mouse libraries. We have also created a database, accessible at http://134.174.53.82/Cepko/, that is searchable by gene name, SAGE tag sequence, accession number, genome location, or UniGene number. It displays all SAGE tags and their levels, as well as ISH images (see below).

The accuracy of the SAGE data was assessed by comparing the 15,268 SAGE tags from E14.5 retina to an unnormalized and unsubtracted set of 15,268 ESTs generated by another research group from E14.5 mouse retina of a different strain (Mu et al. 2001). An r-value of 0.65 (see Figure S1) was obtained that compares well with SAGE expression profiles obtained in similar tissues but from different individuals that were not strain-matched (Blackshaw et al. 2003).

### Analysis of SAGE Tag Expression Patterns in Developing Retina Using Cluster Analysis

**Figure 1:**
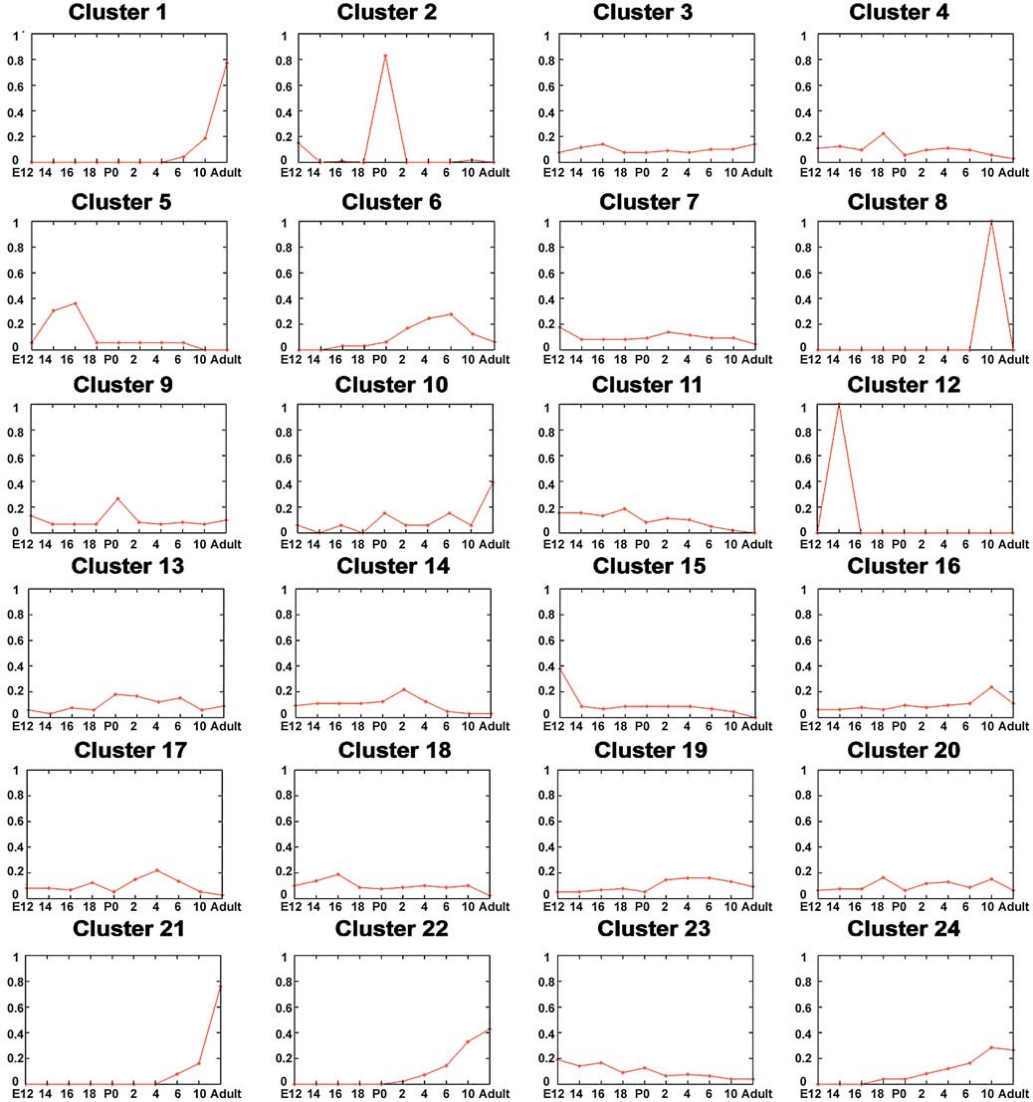
Median Plot of SAGE Tag *K-*Means Cluster Analysis Using 24 Clusters Tags present at greater than 0.1% in one or more of the ten wild-type total retina libraries are considered. SAGE libraries are plotted on the x-axis, and tag abundance, plotted as a fraction of the total tags for a gene in the library in question, is shown on the y-axis. A full list of tags and their abundance levels used for the analysis is detailed in Table S3.

In order to determine whether the temporal pattern of a gene's expression during retinal development might predict its cellular site of expression or its molecular function, clusters of coexpressed genes were assembled. The ten libraries obtained from wild-type total retina were analyzed by cluster analysis using a new Poisson model–based *k*-means algorithm designed specifically for SAGE data (Cai et al. 2004) (see Materials and Methods for a full description of the algorithm and the protocols used). The results for a 24-cluster analysis are shown graphically in Figure 1. Table 1 provides a list of previously characterized genes corresponding to tags within these clusters, the number of genes associated with tags within each cluster that were tested via ISH, and select functional categories of genes that were enriched in specific clusters. Table S3 lists all SAGE tags used in the analysis and their corresponding cluster assignments.

**Table 1:**
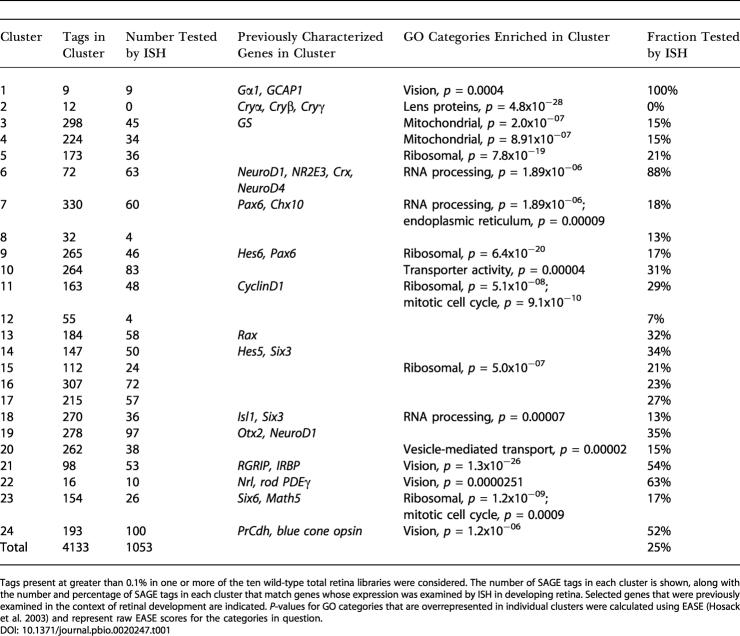
Summary of SAGE Tag *K-*Means Cluster Data

Tags present at greater than 0.1% in one or more of the ten wild-type total retina libraries were considered. The number of SAGE tags in each cluster is shown, along with the number and percentage of SAGE tags in each cluster that match genes whose expression was examined by ISH in developing retina. Selected genes that were previously examined in the context of retinal development are indicated. *P*-values for GO categories that are overrepresented in individual clusters were calculated using EASE (Hosack et al. 2003) and represent raw EASE scores for the categories in question

Virtually every gene previously reported to regulate retinal development was detected in this analysis and showed dynamic expression during development. Several of these transcripts were found at high levels during their period of peak expression. For instance, *NeuroD1*—which regulates rod photoreceptor survival, as well as possibly rod differentiation (Morrow et al. 1999; Wang et al. 2001)—makes up 0.34% of all retinal mRNA at P4.5. In the case of genes previously shown to be required for production of certain cell types in the developing retina, such as *Ath5* and *Chx10*—which are required for ganglion cell and bipolar neurons , respectively (Burmeister et al. 1996; Morrow et al. 1999; Brown et al. 2001; Wang et al. 2001)—peak expression typically occurred around or just after the peak time of exit from mitosis for that cell type.

Certain functional categories of genes were highly overrepresented in a number of SAGE tag clusters. Ribosomal proteins, which typically showed higher expression early in development, were highly enriched in clusters 5, 9, 10, 15, and 23 (Table 1)—clusters that also were enriched for cell cycle regulators (particularly clusters 10 and 23). Mitochondrial proteins, by contrast, were concentrated in clusters 4 and 5. Cluster 2 consisted entirely of crystallins, which may be due to contamination by lens tissue in the E12.5 and P0.5 libraries. Phototransduction genes, on the other hand, were found to be concentrated in the late-onset clusters 1, 21, 22, and 24. Genes representing a number of other functional categories also were enriched in specific clusters, although the reasons in these cases are not clear. Examples of this include the concentration of genes involved in RNA processing in clusters 6 and 7, genes coding membrane transporters in cluster 10, and genes that are involved in vesicle-mediated transport in cluster 20.

### Large-Scale ISH of Dynamically Expressed Genes

Genes identified by SAGE were chosen for analysis via ISH by focusing on genes that showed dynamic expression by *k-*means cluster analysis using Euclidean distance, and some degree of retinal enrichment (i.e., genes were expressed at lower levels in nonretinal SAGE libraries—see Table S2). Within this data set, genes whose presumptive function suggested that they might regulate cell fate choice (e.g., transcription factors, growth factors and their receptors, etc.) received highest priority for testing, although many genes of unknown function with developmentally dynamic expression also were tested. See Table S4 for the Gene Ontology Consortium (GO) classification of each probe tested. The analysis was restricted to genes represented by at least 0.1% of total SAGE TAGS in at least one of the retinal libraries, so as to control for sampling variability and to allow for ready detection via ISH. (Exceptions were made for a number of transcription factors and other genes of potentially major functional interest.) This abundance threshold was met by 4,133 tags. Probes corresponding to 1,051 of these tags were tested via ISH. This total included the 346 candidate photoreceptor-enriched genes tested in our previous work (Blackshaw et al. 2001), as well as 37 previously characterized retinal genes that served as positive controls for ISH and to allow clarification of cellular expression patterns. Retinal expression was examined at every time point used for SAGE (see Materials and Methods for details). See Table S5 for a full list of the cellular expression data for each probe in the retina, along with the accession number of the cDNA used to generate each probe used for ISH. See also http://134.174.53.82/cepko/ for images of all of the ISH data.

### Classification of Cellular Gene Expression Patterns in the Developing Retina

The laminar structure of the retina makes it relatively straightforward to assign a tentative identity to cells expressing a given gene. During early stages of retinal development, the outer neuroblastic layer (ONBL) consists almost entirely of mitotic progenitor cells, while newborn neurons (mostly consisting of amacrine and ganglion cells) reside in the inner neuroblastic layer (INBL). The position of mitotic progenitors within the ONBL varies depending upon their progress through the cell cycle, with S phase cells being found on the vitreal side of the ONBL near the border with the INBL and M-phase cells being found on the scleral side of the ONBL abutting the retinal pigment epithelium (Young 1985a, 1985b). Around the time of birth, immature photoreceptors occupy the outer portion of the ONBL. They are comingled with mitotic cells of the G2, M, and G1 phases of the cell cycle, while the S phase mitotic progenitors are in the vitreal side of the ONBL. Finally, by P6, most retinal cells occupy their final positions within the retina. Rod and cone photoreceptors occupy the ONL. Bipolar neuron cell bodies occupy the scleral portion of the inner nuclear layer (INL); the cell bodies of Müller glia occupy a strip in the center of the INL; and amacrine cell bodies are found in the vitreal portion of the INL. The ganglion cell layer (GCL) contains both ganglion cells and a displaced amacrine cells.

In the developing retina, expression in the scleral and vitreal portions of both the ONBL and INBL were scored separately, along with whether the gene in question was expressed in all or only a subset of cells in the layer in question. In the case of the adult retina, cell identity in wild-type animals could be scored readily by laminar position of the cells expressing the gene of interest (Rodiek 1998), and thus the identity of expressing cells was scored directly.

Extracting order from the diversity of gene expression patterns observed in the developing nervous system can be a daunting task. It is not obvious how best to generate a useful taxonomy of these expression patterns. In tackling this problem, we found it useful to classify cellular expression patterns of genes both by eye and by clustering software. Both methods have specific advantages—user classification more readily identifies rare but distinct patterns, while machine-based clustering allows more flexibility with respect to cluster number and appears to better accommodate classification of intermediate patterns. All classifications were based on the location of the ISH signal within the retinal layers over time during development. Table S6 contains the full list of expression patterns generated by visual inspection, and Table S7 has the full list of cellular expression clusters generated by clustering software. See Materials and Methods for more details on how these data were generated.

Comparison of the user-annotated and machine-generated clusters demonstrated fairly strong similarities between the two sets of clusters (Table S8), although genes placed in a single category by user annotation were invariably grouped into larger clusters by clustering software. On the other hand, genes in certain large clusters generated by user annotation—such as panretinal, *TRAP2*-like, and *Nlk*-like (see Table S6)—were dispersed among many clusters in the machine-generated data sets, with placement within particular clusters varying with replicate program runs. Genes in these categories were expressed at some level in most cells of the developing and mature retina. This variability likely reflects the relative lack of specificity of the expression pattern in these clusters. The finding that most of the highly cell-specific clusters identified by user annotation were readily distinguished by the clustering software supports this hypothesis (Table S8).

### Using SAGE Data to Predict Cellular Expression Patterns in Developing Retina

Temporal changes in gene expression as measured by SAGE turn out to be a useful but inexact method of predicting cellular expression patterns of genes within the retina. While no SAGE cluster was invariably associated with a given cellular expression pattern, genes in certain late-onset SAGE clusters (e.g., clusters 1 and 22) were highly likely to be expressed in developing rods. In the case of early-onset gene expression patterns, which would likely be expressed in retinal progenitor cells, comparison to a microarray-based study could be made. Microarray profiling data of 4N progenitor enriched versus 2N cells has led to the identification of a number of these genes as being enriched in 4N progenitor cells (Livesey et al. 2004). These genes were concentrated in a limited number of SAGE tag clusters (particularly clusters 5, 15, and 23), but were largely absent from clusters that showed a perinatal peak in expression (such as cluster 6), which were enriched for genes expressed in developing rods, bipolars, and amacrine cells (see Table S9 for a full breakdown of 4N-enriched genes by SAGE tag cluster).

In general, the temporal expression pattern observed in a given SAGE tag cluster was accurately reflected by the ISH data, although precise prediction of cellular expression patterns based on cluster data were not achieved. Clusters that showed postnatal peaks in expression, such as cluster 6, could contain a great diversity of cellular expression patterns, yet still be enriched for genes that showed strong expression in specific cell types that were differentiating. Table S10, which details the percentage of tags in a given cluster that represent each specific user-annotated expression pattern, can serve as a starting point for predicting the probability that a gene matching a given SAGE tag will show a given cellular expression pattern in the developing retina.

The expression clusters—whether generated by user annotation or clustering software—at best represent a lower limit to the number of distinct expression patterns within the developing retina. Although the number of distinct types of cells in the developing retina is not known, it is undoubtedly high (MacNeil and Masland 1998). Particularly when considering genes expressed in subsets of cells in the ONBL, or subsets of developing amacrine cells, the level of resolution of our ISH-based screen does not allow one to distinguish many of the more complex patterns. Techniques such as multiple-probe fluorescence-based ISH (Levsky et al. 2002) and single-cell microarray analysis (Tietjen et al. 2003) will be required to resolve such questions as whether individual cells coexpress genes that display complex expression patterns.

One interesting and potentially useful finding from the SAGE cluster data is that genes known to have highly selective cell-specific expression within a single retinal cell type could show different times of onset of expression. For instance, there is heterogeneity in the time of onset of expression among the genes that mediate rod phototransduction, a feature that has previously been reported in ferret retina (Johnson et al. 2001). Phototransduction genes were found in four different clusters (see Table 1), with genes such as *RPGRIP* showing comparatively early onset of expression, followed by the progressively later onset timesof *rod arrestin, rhodopsin,* and, finally, *Gα1* and *GCAP1* (see Table S11 for a full list of tags corresponding to these genes). ISH confirmed the accuracy of the SAGE data for these onset times (see Figure S2). This heterogeneity of the time of onset of expression is observed for terminal differentiation markers of every cell type studied in the retina, as well as for markers of subsets of mitotic progenitor cells (see http://134.174.53.82/cepko/ for the full set of ISH data). Such profiles could be explored for the possibility of control by cascades of transcription factors.

### Gene Expression Patterns Define Subsets of Retinal Progenitor Cells

Recent studies in systems as diverse as *Drosophila* neuroblast specification and the specification of neural-crest-derived cells (Anderson 1999; Isshiki et al. 2001; Pearson and Doe 2003) have demonstrated the role of temporal changes in gene expression in the specification of neural cells. With respect to the retina, the competence model as originally proposed predicted that mitotic progenitor cells would show both temporal changes in gene expression across broad sets of retinal progenitors, and expression of selected genes in specific subsets of progenitor cells at a given time (Cepko et al. 1996).

**Figure 2:**
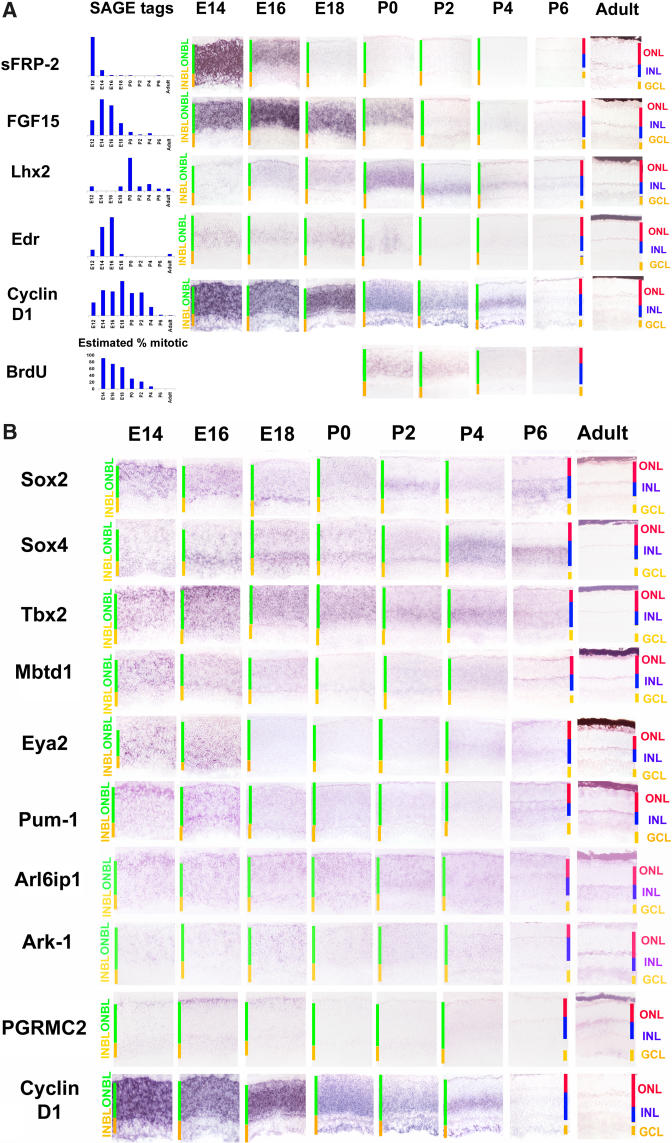
Genes Expressed in Subsets of Mitotic Progenitors (A) Genes expressed in temporally distinct subsets of progenitors. The first column shows relative SAGE tag levels for each gene under consideration. The UniGene identities and common names of the genes in question are *Mm.19155/sFrp2, Mm.3904/Fgf15, Mm.142856/Lhx2, Mm.35829/Edr,* and *Mm.22288/cyclin D1*. The sections for ISH and BrdU shown here were taken from near the center of the retina at the developmental times shown. Mice were albino Swiss Websters except in the case of the adults, which were pigmented C57B/6. See Table S5 for a full list of probes used. Cellular laminae of both the developing and mature retina are indicated with colored bars. All pictures were taken at 200x. The graph plotting the fraction of mitotic cells in the retina adjacent to the BrdU staining is an estimate based on data from both rat and mouse (Young 1985a, 1985b; Alexiades and Cepko. 1996). (B) Spatially heterogeneous ONBL. Genes that were expressed in spatial subsets of cells in the prenatal ONBL are shown. The genes shown are *Mm.4541/Sox2, Mm.18789/Sox4, Mm.4605/Tbx2, Mm.29067/Mbtd1, Mm.2229/Eya2, Mm.34701/Pum1, Mm.29924/Arl6ip1, Mm.11738/Ark-1, Mm.40321/Pgrmc2,* and *Mm.22288/cyclin D1*. Sections were from central retina. Cellular laminae of both the developing and mature retina are indicated with colored bars. All pictures were taken at 200x. See Table S5 for a full list of probes used.

We have identified a number of genes that show temporally restricted expression in early ONBL. By analyzing the expression of a large number of genes that were highly expressed early in development (particularly in SAGE tag clusters 5, 11, and 15), a number of genes that are expressed in broad but temporally restricted subsets of mitotic progenitor cells were identified (Figure 2A). *sFrp2* RNA was found to be broadly expressed in the ONBL until E16, after which it rapidly decreased, a pattern that corresponded well with its SAGE tag levels. Expression of *Fgf15* and *Edr* RNA was seen to persist longer, but neither was easily detected after P0, at which time both *cyclin D1* mRNA—a recognized marker of mitotic progenitor cells in the retina (Sicinski et al. 1995; Ma et al. 1998)—and BrdU labeling were still readily detectable in the central retina. *Edr* RNA showed an unusual patchy distribution in the ONBL at P0—a pattern that was not detected for any other gene tested and has not been previously reported. *Lhx2,* by contrast, was weakly expressed in subsets of cells in the ONBL until P0, when it was dramatically and transiently upregulated throughout the ONBL. Microarray analysis of 4N versus 2N retinal cells at E16 indicates that both *sFrp2* and *Lhx2* are enriched in 4N mitotic progenitor cells (Livesey et al. 2004).

To further investigate the expression of these genes in mitotic progenitor cells, ISH was performed on dissociated retinal cells in conjunction with ^3^H thymidine labeling at E14, E16, and P0 (Table 2). A substantially lower fraction of double-labeled cells for *Fgf15* at P0 relative to earlier time points was observed, while *sFrp2* labeling was absent at birth and substantially lower at E16 than at E14.

**Table 2:**
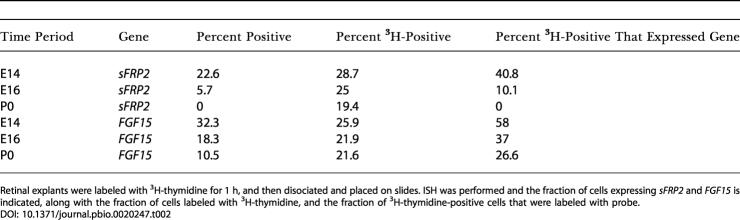
Fraction of Progenitor Cells Expressing *sFRP2* and *FGF15* Decreased as Development Proceeded

Retinal explants were labeled with ^3^H-thymidine for 1 h, and then disociated and placed on slides. ISH was performed and the fraction of cells expressing *sFRP2* and *FGF15* is indicated, along with the fraction of cells labeled with ^3^H-thymidine, and the fraction of ^3^H-thymidine-positive cells that were labeled with probe

A limited number of genes have previously been reported as expressed in subsets of mitotic retinal progenitor cells, including genes such as *Ath5,* and have been shown to be required for retinal ganglion cell development (Brown et al. 2001; Wang et al. 2001). We identified a large number of genes that showed selective expression at certain times during development in relatively small subsets of cells in the ONBL (Figure 2B). These include a large number of known and putative transcription factors, such as *Sox2, Sox4, Tbx2, Eya2* and *Mbtd1* (a novel polycomb family member), along with many genes of other functional classes. Particularly intriguing is the early and transient expression of *Pum1,* a mammalian homolog of the *pumilio* gene, which has been shown to mediate asymmetric mRNA distribution in *Drosophila* (Micklem 1995). Many of these genes showed highly dynamic expression during development—rapidly shifting their cellular expression patterns in the course of a few days, as in the case of *Pum1* and *Sox2,* or being expressed for only a few days, as in the case of *Eya2* and *Pgrmc2*. In some cases, these subsets were scattered throughout the ONBL, such as *Eya2* at E14, while for other genes, such as *Pum1* and *Pgrmc2,* expression was in only the scleral portion of the ONBL, suggesting that these genes may show strongest expression near M phase in retinal progenitor cells.

From these data, it is difficult to determine whether most of these genes were expressed in cycling progenitor cells or cells that have newly exited from mitosis, as these two populations are intermingled in the ONBL. However, microarray analysis of 4N versus 2N cells of the early retina (Livesey et al. 2004) has indicated that a number of these genes, such as *Sox2,* are enriched in 4N progenitor cells. See Figure S3 for more examples of genes expressed in subsets of ONBL cells and contrast with Figure S4, which shows genes with broad but selective expression in the ONBL.

The genes that are expressed in subsets of presumptive retinal progenitors include a large number of transcription factors (e.g., *Sox2, Lhx2,* and *Eya2*) as well as signal transduction components. These intrinsically acting factors represent potential candidates for regulating developmental competence and, by analogy with the *Drosophila* retina, may act combinatorially to help specify cell fate (Flores et al. 2000). Furthermore, a number of genes that are expressed in temporal subsets of progenitor cells encode secreted differentiation factors such as *FGF15* and *sFRP2*. Since cell fate choice is determined by the interaction of intrinsic properties and extrinsic factors, these genes are good candidate regulators of cell fate determination.

Strikingly, the temporal expression profile of very few progenitor-enriched cell cycle genes tracked precisely with the fraction of mitotic cells in the retina. Even many well-established markers of mitotic progenitor cells, such as *cyclinD1* and *cdk4* were highly expressed until P2.5 and detectably expressed as late as P6.5—long after the fraction of mitotic cells in the retina had decreased drastically (Figure 2A). These data imply that expression of these genes frequently persists after the end of mitosis. In addition, one might have predicted that the levels of cell cycle regulators would be highest at the earliest time point analyzed (E12.5), when the percentage of mitotic cells was highest. However, we found that progenitor-enriched genes such as *cyclinD1* and *cdk4* often had RNA levels that peaked around P0.5. This observation suggests that the number of mRNA molecules per cell for many of the genes that mediate mitotic activity increases as development proceeds. The functional significance of these findings is unclear, although a number of features of retinal progenitor cells change over the course of development, including the length of the cell cycle (Young 1985a; Alexiades and Cepko 1996) and the probability of producing progeny that are no longer mitotic (Livesey and Cepko 2001).

### Genes Expressed in Immature Differentiating Retinal Cell Subtypes

**Figure 3:**
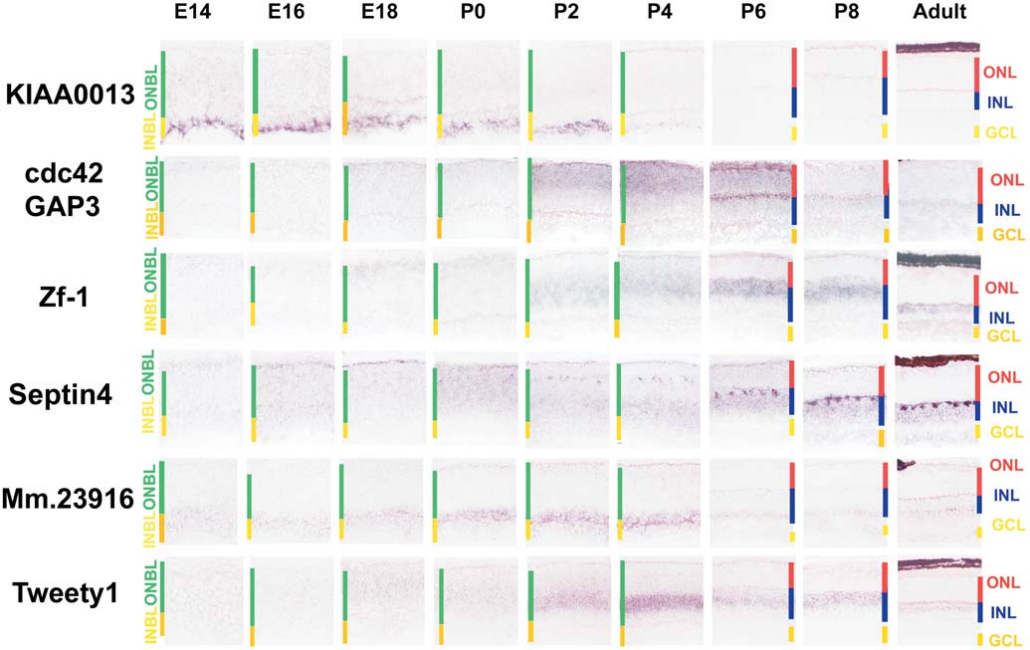
Precursor Patterns for Major Retinal Cell Types Genes that are selectively expressed in immature subtypes of retinal cells. From the top, the differentiating cell types that express the genes in question are ganglion cells *(Mm.45753/KIAA0013),* rod photoreceptors *(Mm.103742/Cdc42GAP),* bipolar cells *(Mm.29496/Zf-1),* horizontal cells *(Mm.2214 /septin 4),* amacrine cells *(Mm.23916),* and Müller glia *(Mm.29729/Tweety1).* Sections were from central retina. Cellular laminae of both the developing and mature retina are indicated with colored bars. All pictures were taken at 200x. See Table S5 for a full list of probes used.

One characteristic expression pattern of genes likely to be involved in cell fate specification and/or the early steps of the differentiation process is restriction to newly postmitotic cells and cells actively undergoing differentiation. Many of the genes demonstrated to show such expression in developing retina, such as *Crx, Nrl,* and *NR2E3* (Furukawa et al. 1997a, 1997b; Chen et al. 1997; Haider et al. 2001; Mears et al. 2001) have been shown to play an active role in regulating cell differentiation. We have identified genes that are selectively expressed in immature postmitotic retinal cells of every major class, with the exception of cone photoreceptors, greatly expanding the set of genes known to be selectively expressed in immature retinal precursor cells (Figure 3). *KIAA0013,* an uncharacterized RhoGAP, was found to be expressed exclusively in immature ganglion cells, and only expressed detectably outside in limited subsets of developing neurons, such as Cajal-Retzius cells of the developing cerebral cortex, and the developing thymus. *Cdc42GAP* was found to be strongly and transiently expressed in newly postmitotic rods, while the leucine zipper transcription factor *Zf-1* was expressed in presumptive bipolar cells. *Septin 4* was found to be selectively and persistently expressed in developing horizontal cells, while *Mm.23916,* a novel dual-specificity protein phosphatase, was found to be expressed selectively in immature amacrine cells. Finally *Tweety1,* an unconventional chloride channel (Suzuki and Mizuno 2004) was strongly expressed in newly postmitotic Müller glia. Along with genes whose cellular expression could be clearly identified visually, a number of genes with strong but transient expression in undefined subsets of cells of the neonatal retina were observed. Expression of these genes persisted after the end of mitosis in the central retina (see Figure 2A), so at least some of the cells that express them must be postmitotic. Genes in this category include *inhibin βB, brain fatty acid binding protein 7, BMP7,* the transcription factor *Sal3,* and the orphan neurotransmitter transporter *NTT7*(see Figure S5).

### Genes Expressed in Developing Photoreceptor Cells

**Figure 4:**
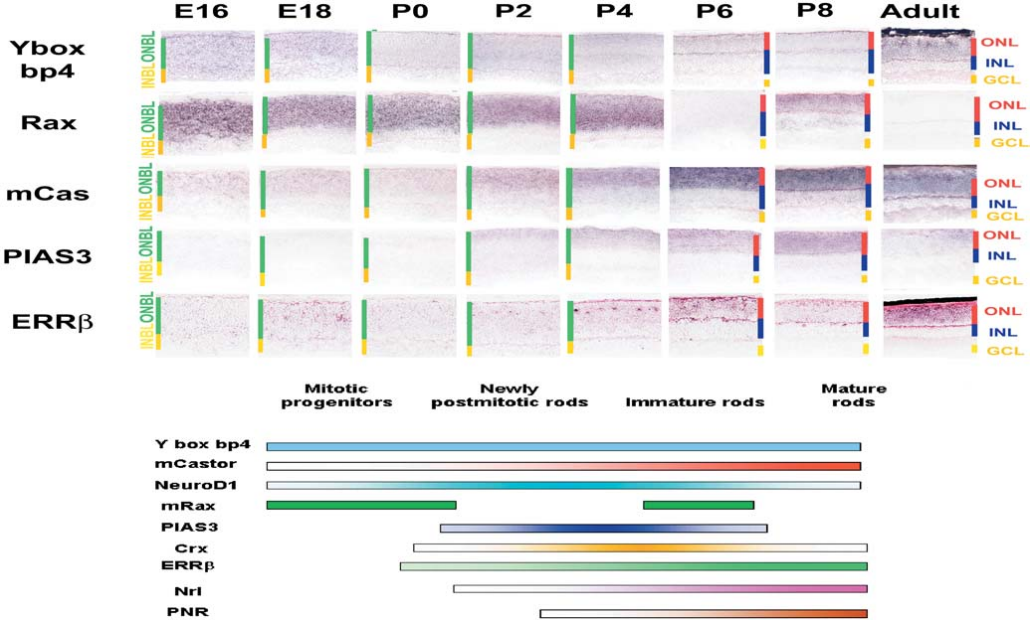
Transcription Factor Cascade in Photoreceptor Development Transcription factors that are selectively expressed in developing rods (and possibly cones as well) are shown. The schematic diagram integrates gene expression data from previously identified photoreceptor-enriched transcription factors and from genes explored in this study. The genes shown are *Mm.193526/Yboxbp4, Mm.3499/Rax, Mm. 89623/mCas, Mm.1635/PIAS3,* and *Mm.235550/ERRβ*. See Figure S6 for images of the developmental expression patterns of previously characterized transcription factors. Sections were from central retina. Cellular laminae of both the developing and mature retina are indicated with colored bars. All pictures were taken at 200x. See Table S5 for a full list of probes used.

Rod photoreceptors make up 70% of cells in the retina (Young et al. 1985b; Jeon et al. 1998). The SAGE-derived expression profile of genes selectively expressed in developing rods is thus more comprehensive than that of other cell types. Based on the ISH data and aided by our SAGE study of mature tissue (Blackshaw et al. 2001), as well as previous reports of mutant mice lacking transcription factors known to be important for rod development, a model of a temporal order of transcription factor expression during rod development was made (Figure 4). Transcription factors known to be involved in cell fate specification sometimes show broad expression in mitotic progenitor cells and persistent expression in mature cell types (e.g., Liu et al. 1994; Belecky-Adams et al. 1997; Livesey and Cepko 2001). We observed a number of genes that were expressed in early ONBL from E16 on, with expression persisting in mature photoreceptors, such as *Yboxbp4*. A similar pattern were seen for the mouse ortholog of the Drosophila castor gene, though this gene was observed in a more restricted subset of cells in the ONBL at E16, and for the orphan nuclear receptor *ERRβ,* although this gene had relatively lower expression prenatally and had pronounced expression in an undefined subset of cells in the immature photoreceptor layer during the first postnatal week.

In contrast to being expressed in mitotic cells as well as differentiating photoreceptor cells, a number of transcription factors were selectively expressed in postmitotic but immature photoreceptors. The *Rax* homeodomain factor showed, as has been previously reported (Furukawa et al. 1997a; Mathers et al. 1997), strong expression in mitotic progenitor cells in the ONBL that vanished with the end of mitosis. However, expression transiently reappeared in immature photoreceptors at P8. This situation is analogous to that seen in a number of other vertebrates, in which a duplication of the ancestral *Rax* gene has resulted in *Rax* genes with distinct expression in photoreceptor and progenitor cells (Chuang et al. 1999; Chen and Cepko 2002). *PIAS3,* which encodes a SUMO lyase that directly regulates the activity of a broad subset of transcription factors (Kotaja et al. 2002; Haider et al. 2001), was strongly and selectively expressed only in developing photoreceptors, with expression beginning at E18, peaking at P8, and largely fading away in the adult, a pattern that in many respects is reminiscent of *Crx* (see Figure S6). In contrast to these patterns, *Nrl* and *NR2E3* showed no detectable expression prenatally, and showed peak expression around P6. Somewhat surprisingly, the RNAs for many of these transcription factors is enriched in the inner segments of photoreceptors, as are a large fraction of the other photoreceptor-enriched genes characterized in this study, a finding that is in line with our earlier work (Blackshaw, et al. 2001). The functional significance of this remains unclear.

In addition to transcription factors, other functional classes of genes, including genes of unknown function, were expressed in developing photoreceptors, with strongest expression typically found in the first postnatal week (Figure S7). In some cases, these genes fall into pathways known to regulate rod differentiation. Both *PIAS3* and the multifunctional protein *Hrs* (Chung et al. 1997; Scoles et al. 2002) selectively inhibit *STAT3,* and thus possibly inhibit the action of ciliary-derived neurotrophic factor, a factor that has been shown to inhibit rod differentiation in rodents (Ezzeddine et al. 1997; Kirsch et al. 1998; Schulz-Key et al. 2002). *Cdc42GAP* expression (see Figure 2) may mediate the polarization and initiation of outer segment formation taking place in photoreceptors at this time (Nobes and Hall 1999). In other cases, genes newly identified as selectively expressed in developing photoreceptors imply the existence of novel facets of photoreceptor development. The expression of synaptic vesicle protein *Cpx2* suggests that developing photoreceptors may be actively secreting some developmentally relevant signal, while the expression of *Hrs* also potentially suggests high levels of regulated endocytosis and destruction of unknown extracellular proteins (Lu et al. 2003). The expression of the previously uncharacterized tumor necrosis factor family member *Tnfsf13* and A20-like signal transduction components such as *TRABID* and *Fln29* suggest an unexplored role for this pathway in normal photoreceptor development.

### Genes Expressed in Developing Interneurons of the INL

Many genes were selectively expressed in the other, nonphotoreceptor retinal cell types during development. A temporal sequence of transcription factors was observed in bipolar cells as they differentiated (Figure S8). The homeodomain factor *Lhx4,* and the uncharacterized leucine-zipper protein *Zf-1* (see Figure 2), showed expression at E16 in the ONBL, with expression continuing postnatally and persisting in adult bipolar cells. *Zfh4* was expressed in developing amacrine cells and in subsets of cells in the ONBL prior to P4, and was robustly and transiently expressed in bipolar cells, with peak expression at P6. The relatively late-onset *Dbp* was first seen in the second postnatal week across the INL. *Chx10,* as has been previously reported (Liu et al. 1994), and *Gli5* were broadly expressed across the ONBL prior to P4, at which point they both showed elevated expression in developing bipolar cells. Microarray analysis confirmed that both of these genes are expressed in mitotic progenitor cells (Livesey et al. 2004). Possible downstream targets of these transcription factors include previously uncharacterized cell adhesion molecules such as the Ig-superfamily member *Mm.41284,* kinases such as *Prkcl,* and the putative growth factor receptor *SEZ-6*. Furthermore, despite the fact that they comprise only 0.3% of the cells in the adult retina, genes that are highly enriched in both developing and mature horizontal cells (Figure S9), such as the GTPase regulator *Borg4,* were found.

Many genes tested by ISH were selectively expressed in developing amacrine cells (Figure S10). The expression patterns were tremendously diverse, a fact that may reflect the reported extensive heterogeneity among amacrine cell subtypes (MacNeil and Masland 1998). Certain genes, such as the kinase *Unc51-like-1, ArfGAP,* and the orphan G-protein-coupled receptor *Mm.6393,* were found to be expressed both in immature amacrine cells and in subsets of cells in the ONBL, particularly in the region of the ONBL that comprises the outer or scleral surface, where M phase mitotic progenitor cells are localized. Cytoskeletal-associated kinases such as *Unc51-like-1,* and small GTPases such as *ArfGAP,* may play a role in neurite extension or process formation. Additionally, the expression of neuropeptide receptors such as *Mm.6393* in the ONBL before mature neural circuits have formed fits with data from other parts of the developing CNS showing early expression of neurotransmitter receptors and suggesting that neurotransmitters may act on mitotic progenitor cells to regulate cell cycle or cell fate specification (Rueda et al. 2002; Ohtani et al. 2003). Similarly, recent work from our laboratory on the role of glycine receptors in the formation of rod photoreceptors (Young and Cepko 2004) confirms such predictions for at least one such receptor.

Other genes, such as *syntrophin-associated kinase* and the novel dual-specificity phosphatase *Mm.23916,* were confined to immature amacrines only. *Syntrophin-associated kinase,* in particular, may regulate maturation of synaptic connections (Lumeng et al. 1999). Others genes, such as *necdin,* the basic helix-loop-helix transcription factor *Nhlh2,* and the novel PLC isoform *Mm.215653,* showed complex and often biphasic patterns. The Slit receptor *robo3* was strongly and transiently expressed in the first postnatal week in a single sublamina within the INBL, perhaps corresponding to a single subtype of developing amacrine cells. A role for Slit-Robo signaling in regulating cortical dendrite maturation has been demonstrated (Whitford et al. 2002), and these data suggest such a mechanism may be at work in regulating subtype-specific amacrine cell laminae formation in the retina. *Neuropeptide Y* was strongly and transiently expressed in a subset of amacrine and horizontal cells towards the end of the first postnatal week, with expression dropping dramatically in the adult—suggesting a possible role for this factor in the formation of mature retinal circuitry. Finally *Mm.41638,* which is weakly homologous to a lysosomal membrane protein, was expressed solely in postnatal amacrine cells, though expression remained in a more restricted subset of amacrine cells in the adult.

### Müller Glia Are Highly Similar to Retinal Progenitor Cells

**Figure 5:**
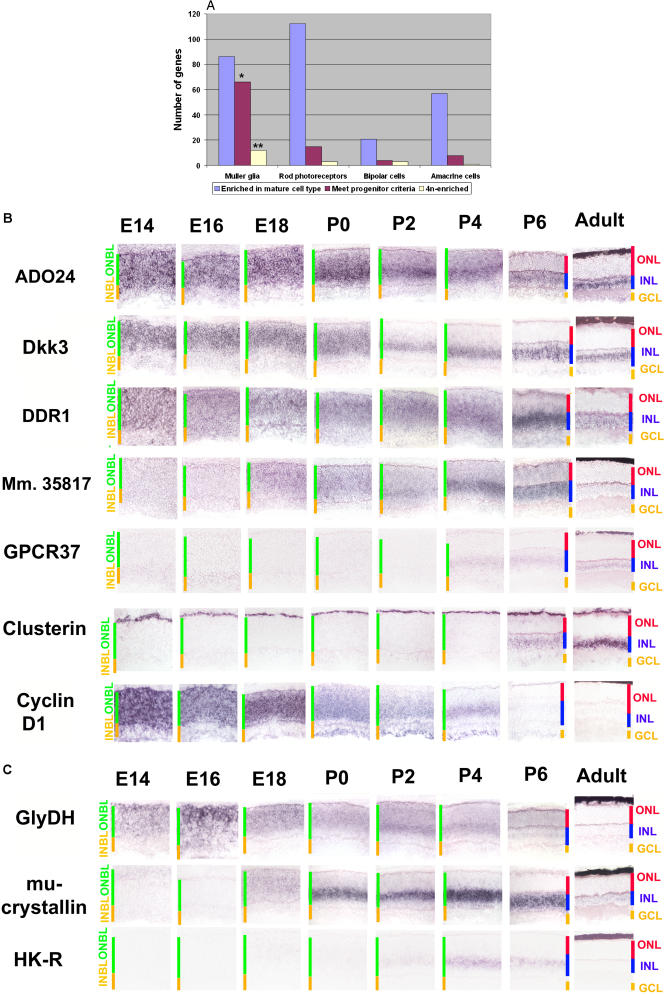
Müller-Glia-Enriched Genes (A) Müller-glia-enriched genes show stronger expression in retinal progenitors than do genes enriched in other postnatally born cell types. See Materials and Methods for details of how progenitor-enriched and cell-specific expression patterns were determined, and *p*-values for progenitor-enrichment of genes that are cell type–specific in the mature retina were calculated. Data on 4N-enriched transcripts were obtained from Livesey et al. (2004). Numbers for each value are as follows. For *N,* the number of cell-enriched genes, *N*_MG_ = 86, *N*_Pr_ = 112, *N*_BC_ = 21, and *N*_AC_ = 57. For *I,* the number of genes that show retinal progenitor-enriched patterns by ISH, *I*_total_ = 180, *I*_MG_ = 66, *I*_PR_ = 15, *I*_BC_ = 4, and *I*_AC_ = 8. For *M,* the number of genes enriched in 4N retinal progenitor cells that were tested by ISH in adult retina, *M*_total_ = 28, *M*_MG_ = 12, *M*_PR_ = 3, *M*_BC_ = 3, and *M*_AC_ = 1. *, *p* < 10^−13^; **, *p* < 0.0001. (B) Müller-glia-enriched genes show strong expression in mitotic progenitors. The genes shown are: *Mm.26062/ADO24, Mm.55143/Dkk3, Mm.5021/DDR1, Mm.35817, Mm.20465/GPCR37, Mm.200608/clusterin,* and *Mm.22288/cyclin D1*. Sections were from central retina. Cellular laminae of both the developing and mature retina are indicated with colored bars. All pictures were taken at 200x. See Table S5 for a full list of probes used. (C) Dynamic expression of metabolic genes in developing retina. Metabolic enzymes are often selectively expressed in mitotic progenitors and developing Müller glia. The genes shown are *Mm.27953/glycine decarboxylase, Mm.9114/mu-crystallin,* and *Mm.213213/HK-R*. Cellular laminae of both the developing and mature retina are indicated with colored bars. Sections were from central retina. All pictures were taken at 200x. See Table S5 for a full list of probes used.

Genes selectively expressed in Müller glia share a number of defining features. Mitotic retinal progenitor cells and Müller glia showed a great degree of transcriptional overlap—far more so than other retinal cells that differentiate postnatally. Of the genes identified as being specifically expressed in Müller glia after the first postnatal week, 68% were found to be enriched in mitotic progenitor cells based on their ISH pattern, in contrast to only 14% of photoreceptor-specific genes (Figure 5A). Of the genes identified as enriched in 4N progenitor cells by micorarray analysis (Livesey et al. 2004) that were tested by ISH in adult retina, 43% were enriched in Müller glia, compared to 11% that were enriched in photoreceptors.

Typical expression patterns for Müller-glia-enriched genes are shown Figure 5B. Genes in this category, such as the negative regulator of Wnt signaling *Dkk3,* the collagen receptor *DDR1,* and the endosomal protein *AD024,* were observed to be strongly and broadly expressed across the ONBL throughout development, though expression in the adult was restricted to Müller glia. Microarray analysis suggests that a number of these genes, including *Dkk3* and *DDR1,* are enriched in 4N mitotic progenitor cells (Livesey et al. 2004). A smaller set of genes, such as *Mm.35817, GPCR37,* and *Tweety1* (see Figure 2) were found to be expressed across the ONBL early in development, but showed dramatically and transiently upregulated expression at the end of the first postnatal week as Müller glia began to differentiate. While over two-thirds of Müller-glia-enriched genes showed enriched expression in retinal progenitors relative to other cell types in the developing retina, virtually all Müller-glia-enriched genes were expressed at detectable levels in retinal progenitors (without necessarily being enriched in progenitors). In fact, only two genes that are Müller-specific in the adult—*clusterin* and *carbonic anhydrase 2*—were expressed in mature Müller glia but not detected in mitotic progenitors. However, previous work suggests that *carbonic anhydrase 2* may be expressed in retinal progenitors at levels below our ability to detect (Vardimon et al. 1986), and this may be the case for *clusterin* as well. Additional Müller-glia-enriched genes are shown in Figure S11.

The extensive overlap in gene expression between Müller glia and mitotic progenitor cells raises the question of how closely these two cell types resemble each other at the functional level. Müller glia morphologically resemble mitotic progenitor cells in having apical and basal processes that span the radial dimension of the retina (Rodiek 1998)—a feature that is shared with retinal progenitor cells as well as radial glia of the developing brain, a cell type known to be the cortical progenitor cell (Doetsch 2003). Müller glia are one of the last cell types to exit mitosis (Young 1985b; Reh and Levine 1998), and they are the only cell type in the mature retina that can reenter mitosis following retinal injury (Dyer and Cepko 2000b; Vetter and Moore 2001). Finally, data from chicken suggest that, at least in some birds, Müller glia can be induced to divide and give rise to some types of retinal neurons for a short period of time near the end of retinal development (Fischer and Reh 2001). The question arises, then, as to whether Müller glia are fundamentally multipotent progenitor cells that are quiescent regarding cell division and the production of neurons (Morest and Silver 2003; Walcott and Provis 2003). If they are progenitor cells, they are progenitor cells that have acquired the specialized properties needed for a support role in the mature retina, e.g., neurotransmitter reuptake and structural roles. The few genes that are specifically expressed in mature Müller glia, such as *clusterin,* may be emblematic of such roles. Misexpression in mature Müller glia of genes that are candidates for regulating neuronal production in the postnatal retina, followed by injury-induced division, offers a potential approach for future therapies that might lead to photoreceptor or ganglion cell replacement in diseased retinas by cells derived from Müller glia.

### Prominent Expression of Metabolic Enzymes in Developing Müller Glia

A second notable feature of genes expressed nearly specifically in developing Müller glia is the highly dynamic and cell-specific expression of a number of metabolic enzymes (Figure 5). The novel hexokinase-related gene *HK-R* was selectively expressed in developing Müller glia cells, but not in any other cell in the body examined. *Mu-crystallin,* which does not encode a crystallin in placental mammals but rather an uncharacterized homolog of the bacterial enzyme ornithine cyclodeaminase (Segovia et al. 1997), showed a similar expression pattern in the retina but also was expressed in other developing sensory organs. Glycine decarboxylase was strongly and selectively expressed in retinal progenitor cells, differentiating Müller glia, and to a lesser extent, developing photoreceptors.

The reasons for such high enzymatic activity in development is unclear, although some of these genes may have regulatory functions unconnected to their metabolic roles. For instance, mu-crystallin is also a thyroid hormone binding protein (Vie et al. 1997). Such proteins also may regulate the abundance of small molecules that can act as signals that may be relevant for development. For example, glycine levels may be kept low by glycine decarboxylase so that taurine can bind to and activate the glycine receptor to promote rod differentiation (Young and Cepko 2004). These data point to future directions of research examining the intersection of metabolism and development and suggest the usefulness of supplementing gene expression profiling with metabolomic analysis (Watkins and German 2002).

### Dynamic Expression of Putative Noncoding RNAs in Developing Retina

**Figure 6:**
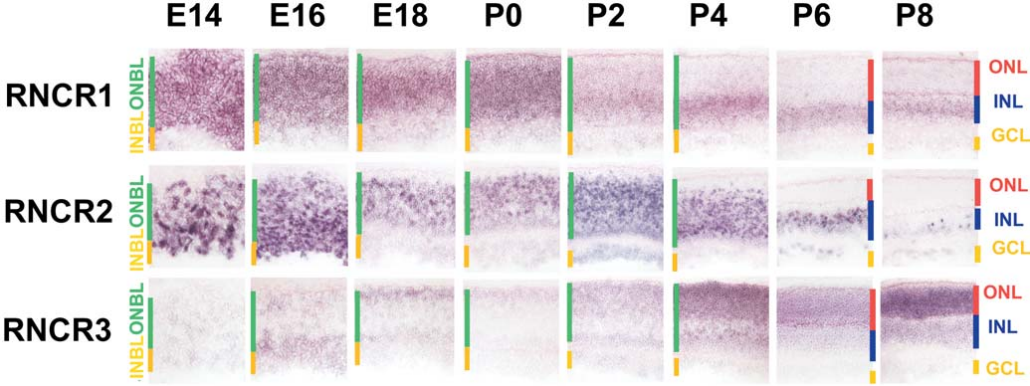
Noncoding RNAs in Retinal Development A number of presumptive noncoding RNAs are strongly expressed in dynamic subsets of retinal progenitor and precursor cells. The transcripts shown are *Mm.150838/ RNCR1, Mm.44854/ RNCR2,* and *Mm.194050/RNCR3*. Sections were from central retina. Cellular laminae of both the developing and mature retina are indicated with colored bars. All pictures were taken at 200x. See Table S5 for a full list of probes used.

A number of RNA transcripts that do not appear to encode proteins were strongly expressed in the developing retina (Figure 6). These transcripts are typically spliced and polyadenylated, but do not encode evolutionarily conserved open reading frames (ORFs), or any ORFs encoding proteins longer than 100 amino acids, while often showing high similarity at the nucleotide level between mouse and human (Numata et al. 2003). Table S12 provides a list of these transcripts. Putative noncoding transcripts that showed developmentally dynamic expression include *retinal noncoding RNA 1 (RNCR1),* which was expressed throughout the ONBL during early development and which was later restricted to Müller glia. It was transcribed in a head-to-head fashion, and largely coexpressed, with *Six3.* This transcript showed extensive alternative splicing, and while one splice form contained a potential ORF of greater than 100 amino acids, no mouse/human conservation of this putative protein was observed, while high similarity was observed at the nucleotide level in other regions of the transcript. *RNCR2 ,* on the other hand, was expressed in a large subset of cells in both the ONBL and INBL prenatally, with expression restricted to the INL and GCL postnatally. ISH signal for *RNCR2* was strongly concentrated in what appeared to be nuclear or perinuclear regions of expressing cells. *RNCR3* was expressed in a steadily increasing subset of cells in the ONBL from E14 and gradually resolved to an adult pattern that was photoreceptor-enriched but present in the inner retina at lower levels.

Although additional assays are required to conclusively demonstrate that these RNAs do not encode functional proteins, there is precedent for this conclusion from recent genomic work. Large-scale EST sequencing efforts from mouse have uncovered up to several thousand putative spliced transcripts that do not appear to encode for proteins (Numata et al. 2003). Likewise, oligonucleotide array experiments using probes that tile individual human chromosomes at high density report substantial transcription from many regions not predicted to have protein-coding genes (Kapranov et al. 2002; Cawley et al. 2004), and suggest that microarray-based expression profiling that uses probes designed only against predicted protein-coding genes may miss a significant fraction of the transcriptome. The functional role of these transcripts is obscure, although noncoding spliced RNAs such as *Xist* and *H19* in mammals and *Rox1* and *Rox2* in *Drosophila* have been implicated in a variety of epigenetic processes (Mattick 2003). The possibility that *RNCR1* might somehow regulate expression of *Six3* or other progenitor-specific transcripts awaits further investigation.

Both *Xist* and *Tsix,* noncoding RNAs that play a crucial role in X-inactivation, were expressed in subsets of cells in the ONBL and INBL early in development, but were expressed strongly and selectively in the INL around the end of the first postnatal week (Figure S12). This finding is quite surprising, given that photoreceptors and ganglion cells do not express these transcripts and would thus appear to escape X-inactivation. Since genetic evidence suggests that this is not the case for either cell type (Reese et al. 1999), our findings implicate the existence of possibilities such as alternate cell-specific pathways of X-inactivation or dramatic cell-specific variations in *Xist* levels required to mediate X-inactivation.

### Expression Profiling and Candidate Gene Analysis

Although we have identified a plethora of transcription factors, growth factors, and signal transduction components, the data do not clearly implicate a known signaling pathway as selectively involved in the differentiation of a given cell type within the retina. For example, negative regulators of Wnt signaling were identified, but these genes display a diversity of cellular expression patterns that cloud a simple model for their action. *Dkk3* and *Nkd1* are expressed broadly in progenitor cells and Müller glia, together with *beta-catenin,* while *sFRP-2* is expressed exclusively in early progenitor cells, and *Nlk* is expressed strongly in postmitotic but immature cells of the postnatal retina. Another approach to the creation of models of pathways that control retinal development is to combine the ISH analysis of genes identified via SAGE with a candidate gene approach, even for genes not identified by SAGE. For example, we examined the expression of all known regulators of Wnt signaling, all fibroblast growth factor receptors, and all Slit and Robo genes whether or not SAGE tags corresponding to these genes were identified. See Table S5 and http://134.174.53.82/cepko/ for a full list of genes and their expression patterns.

### Cell-Specific Gene Expression in the Mature Retina Identifies Candidate Retinal Disease Genes

**Figure 7:**
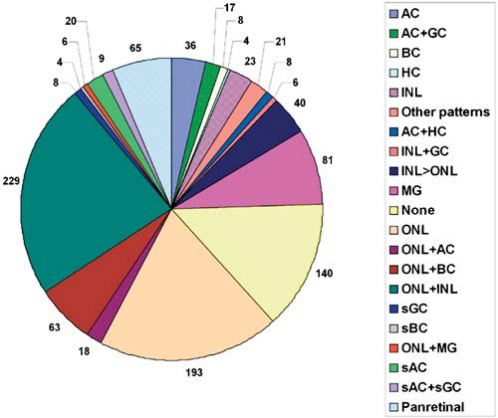
Catalog of Gene Expression in Adult Retina The most commonly observed patterns of gene expression in the adult retina are indicated. Data are taken from Table S5 and cover all genes examined in the adult retina. Genes are placed in a category corresponding to a single cell type if expression is substantially greater in that cell type than in any of the other cell types examined. Genes are placed in categories corresponding to multiple cell types if expression is approximately equal in more than one cell type. The number of genes expressed in photoreceptors and Müller glia differs somewhat from those used in the analysis shown in Figure 5A, since the expression of a large number of photoreceptor-enriched genes was not examined prenatally, and a number of Müller-enriched genes were detectable in Müller glia through the end of the second postnatal week, but not in adult retina. AC, amacrine cells; BC, bipolar cells; GC,ganglion cells; HC, horizontal cells; MG, Müller glia; sAC, subset of amacrine cells; sBC, subset of bipolar cells; sGC, subset of ganglion cells

A molecular catalog of gene expression in the adult retina was assembled with molecular markers for every major class of retinal cell (Figure 7). The catalog of photoreceptor-enriched genes reported in previous work (Blackshaw et al. 2001) was expanded, and a large number of genes expressed in the inner retina were identified. Some of these include genes that mark subsets of amacrine and ganglion cells. Knowledge of which genes show cell-specific expression in the retina can aid in identifying retinal disease genes. The expression of nearly half of all cloned photoreceptor dystrophy genes is selectively enriched in photoreceptors (Blackshaw et al. 2001), while hereditary optic neuropathies have been suggested to be partially mediated by mutations in ganglion-cell-enriched genes (Votruba et al. 1998). Furthermore, a number of other retinal and anterior segment abnormalities result from mutations in genes that are broadly expressed in retinal progenitor cells (Hanson et al. 1999; Ferda Percin et al. 2000). See Table S13 for a full list of the chromosomal locations of the human orthologs of genes examined in this work. This list also contains a full list of mapped but unidentified Mendelian human retinal disease genes and orthologs of photoreceptor-enriched genes identified in this work that lie within those chromosomal intervals. A total of 164 photoreceptor-enriched genes not previously linked to retinal disease were found in chromosomal intervals containing retinal disease loci, representing a total of 42 distinct loci. While photoreceptor-enriched transcripts make up roughly half of all cloned retinal disease genes (Blackshaw et al. 2001), roughly one-third of retinal disease genes are expressed in all cells of the retina, suggesting that it is fruitful to consider such genes when screening candidate disease genes. We find that 22 panretinally expressed genes map within intervals containing unidentified disease genes, representing 16 distinct loci.

### Genomic Approaches to Development

The retina consists of a number of distinct cell types that are relatively well defined morphologically, as well as molecularly. They undergo differentiation in defined intervals and are found in stereotypical locations within the retina. These characteristics allow a fairly straightforward evaluation of the cell-specific expression of genes within the retina. We have coupled SAGE-based expression profiling with large-scale ISH analysis to obtain an atlas of gene expression for the developing and mature retina. This atlas is useful for many purposes—in particular, providing many candidate genes for studies of retinal development and function. SAGE analysis can be nearly comprehensive (Velculescu et al. 1995), but its sensitivity is limited by the number of tags sequenced, the level of expression of a transcript within a given cell, and the abundance of given cell subtypes within a tissue sample. Thus this analysis detected relatively rare cell-specific transcripts primarily for the abundant rod photoreceptors and their precursors, and for genes broadly expressed in retinal progenitor cells. Nonetheless, the catalog does include some genes selectively expressed even in the rarest cell types, such as the horizontal cells (0.3% of all retinal cells; Jeon et al. 1998) and subtypes of ganglion cells, as well as genes expressed selectively in small subsets of cells in the early ONBL.

A recent microarray-based study in developing neural crest screened over 90 candidate genes via ISH (Gammill and Bronner-Fraser 2002), and a recent study using serial stages of embryonic *Drosophila* has analyzed hundreds of genes by such methods (Tomancak et al. 2002). However, while a number of recent studies have used microarray analysis to profile developing neural tissue, large-scale ISH-based validation of genes identified as being expressed in developing CNS by such expression profiling has not yet been conducted. Large-scale ISH studies enhance our ability to interpret expression profiling data, as the precise cellular expression of a gene in heterogeneous tissues of the developing nervous system cannot be inferred reliably from the profiling of bulk tissue.

Other considerations underscore the benefits of verifying primary expression data from expression profiling methods by using other approaches. For instance, several studies describing microarray-based expression profiling of similar starting material have obtained contrasting results for sets of differentially regulated genes (Claridge-Chang et al. 2001; McDonald and Rosbash 2001; Lin et al. 2002; Ivanova et al. 2002; Ramalho-Santos et al. 2002). These may result from either experimental variation among labs or biological variation in gene expression among the samples and individuals tested (Pritchard et al. 2001; Blackshaw et al. 2003), but nonetheless suggest that large-scale verification of expression differences by techniques such as quantitative RT-PCR or ISH would aid interpretation of such differences. Studies that rely on large-scale ISH as an initial screen generate vast amounts of data, but typically have been conducted using sets of identified or random cDNAs without using expression screening to preselect genes that show high or dynamic expression in the tissue of interest (Gawantka et al. 1998; Neidhardt et al. 2000; Kudoh et al. 2001; Thut et al. 2001). Using expression profiling to generate a set of candidate genes for large-scale ISH analysis will increase the probability of testing genes that show enriched or dynamic expression in a tissue of interest.

### Towards a Functional Genomics of Neural Development

The data presented here provide the starting point for medium-throughput functional analysis of the role of many genes in retinal development. The use of in vivo electroporation (Matsuda and Cepko 2004) and plasmid constructs encoding small inhibitory RNAs delivered by electroporation or retroviruses will make possible medium-throughput gain- and loss-of-function studies of gene function in the retina. The identification of a variety of progenitor subtypes and stage-specific precursor markers will enable a deeper interpretation of such studies. Construction of appropriate Cre lines will allow lineage analysis to determine with precision the mature cell types to which subsets of mitotic progenitor cells or posmitotic precursors give rise. Combining the knowledge of cell-specific transcription factors and cell-specific target genes, together with bioinformatic approaches that take advantage of mammalian genome sequence information in a manner like recent efforts in *Drosophila* (Stathopoulos et al. 2002), may allow the characterization of the combinatorial code of *cis*- and *trans*-acting elements that specify mature neuronal identity. We anticipate that similar approaches are likely to be useful in any region of a developing tissue where birthdating studies have been conducted and cell subtypes can be readily identified based on their spatial localization.

## Materials and Methods

### 

#### Generation of SAGE libraries

Isolation of mouse brain and retinal tissue, as well as construction of all SAGE libraries derived from retinal and hypothalamic tissue, was conducted as previously described (Blackshaw et al. 2001). Publicly available mouse libraries used in the analysis include 3T3 fibroblasts (obtained from http://www.sagenet.org), P8 cerebellar granule precursor cells maintained in culture for 24 h (GCPcntr; obtained from http://www.ncbi.nlm.nih.gov/SAGE), P8 cerebellar granule precursor cells maintained in culture and treated with Shh for 24 h (GCP+SHH; obtained from http://www.ncbi.nlm.nih.gov/SAGE), freshly harvested P8 cerebellar granule precursor cells (GC_P8; obtained from http://www.ncbi.nlm.nih.gov/SAGE). Libraries from E15 and P1 cerebral cortex were obtained from Gunnersen, et al. 2002. ). All retinal and hypothalamic SAGE data have been submitted to NCBI, and will be available for download at http://www.ncbi.nlm.nih.gov/SAGE.

#### SAGE data analysis

The SAGE 3.0.1 program (courtesy of Victor Velculescu and Ken Kinzler, Johns Hopkins University School of Medicine, Baltimore, Maryland, United States) was used to extract SAGE tags and eliminate duplicate ditags. Identity of SAGE tags was obtained from the National Center for Biotechnology Information (NCBI) “reliable” tag map set for UniGene (available at http://www.ncbi.nlm.nih.gov/SAGE). UniGene Build 131 of Mus musculus (http://www.ncbi.nlm.nih.gov/UniGene) was used for the mappings. In cases where ISH results for genes matching a “reliable” tag did not match the temporal expression profile for the tag in question, along with all cases of unknown tags (i.e., tags which had no “reliable” tag to gene assignment) that were present at greater than 0.1% of total tags in any one SAGE library, the genes were tested via NCBI BLASTN searching (http://www.ncbi.nlm.nih.gov/BLAST/) against the nr and dbest databases, with Expect threshold set to 100 (Karlin and Altschul 1990). A tag was considered to match a specific transcript if it corresponded to the 3′-most NlaIII site in a given polyadenylated transcript (Velculescu et al. 1995). If no such match was found, tags matching the 3′-most NlaII sites in 5′ reads of retinal-derived MGC cDNAs (Strausberg et al. 2002) were considered to match those transcripts, in cases where no further 3′ sequence information was available for those ESTs. Each tag representing a gene tested by ISH, moreover, was checked by BLASTN using these parameters to verify the accuracy of the NCBI tag-to-gene matches.

Human orthologs of mouse genes were identified through the use of the Homologene data set and verified by BLASTN and/or BLASTX analysis using the NCBI server, or BLAT analysis using the University of California at Santa Cruz genome server (http://genome.ucsc.edu). In cases where no curated ortholog was present in the database, BLASTN analysis against nr, dbest, and htgs databases was used to identify transcripts that showed over 85% sequence conservation over 100 bp and did not match any repeat sequence. The University of California at Santa Cruz genome browser using the October 2003 freeze (http://genome.ucsc.edu/cgi-bin/hgGateway) was used to determine if any transcripts with no obvious coding sequence mapped within 5 kb of the 3′ end of an identified gene and were transcribed in the sense orientation relative to that gene. If so, these were considered to represent novel 3′ ends of that gene. All other data analysis and curation was conducted with Microsoft Excel and Microsoft Access.

#### Tissue section, ISH, and BrdU staining

ISH was conducted as previously described (Blackshaw et al. 2001). For BrdU staining, mice were given a single interperitoneal injection of 37.5 mg/kg BrdU and killed 1 h later. Fresh-frozen sections were used following 15 min fixation in 4% paraformaldehyde. The protocol of BrdU staining was carried out using an anti-BrdU monoclonal antibody (Roche, Basel, Switzerland) and detected using an AP-conjugated secondary antibody, using recommended blocking and washing conditions.

#### Dissociated cell ISH

Retinas were dissected from E14.5, E16.5, and P0 mice and cultured for 1 h in DMEM/10% fetal calf serum containing 5 μCi/ml ^3^H-thymidine. The labeled retinas were dissociated into single cells by incubating for 30 min at 37 °C in 100 units/ml of papain (Worthington Biochemical, Lakewood, New Jersey, United States) in Hank's balanced salt solution (HBSS) containing 10 mM HEPES (pH 7.6), 2.5 mM cysteine, and 0.5 mM EDTA. The suspensions were then gently triturated and incubated with 0.1 mg/ml DNase I for 10 min at 37 °C. The cells were pelleted, washed twice in HBSS, and plated on polyD-lysine-coated glass slides for 15 min at room temperature. Cells were fixed to the slides in 4% paraformaldehyde for 5 min at room temperature, washed twice in PBS, and dehydrated in 100% methanol. For acetylation, probe incubation, and subsequent washings, the in situ protocol detailed herein for tissue sections was used. A tyramide signal amplification system (TSA Plus, PerkinElmer, Wellesey, Massachusetts, United States) combined with an anti-digoxigenin-HRP antibody (Roche) was used according to the manufacturer's instructions to detect the signal. Autoradiographic processing was performed in emulsion (NTB2, Eastman Kodak, Rochester, New York, United States) exactly as previously described (Alexiades and Cepko 1996).

#### Classification of cellular expression data in retina by user-based classification and cluster analysis

Two classification schemes of the patterns of expression over time were developed: human and machine-aided. In the first case, a single observer (S.B.) generated a presumptive minimal classification of expression patterns following visual inspection of each hybridization pattern (see Table S6 for a full list). This subjective classification took into account a relatively informal assessment of signal intensity. This approach yielded a total of 72 distinct patterns, of which 19 contained only a single member. In the second case, laminar expression within the retina was scored on a 0–5 point scale based upon visual inspection for each defined cell type in the prenatal, perinatal, and mature retina, and cluster analysis software was used to perform *k-*means clustering (using Euclidean distance) of cellular expression patterns (see Table S7 for the full data set). As with the cluster analysis of the SAGE data, in order to determine an optimal minimal number of clusters, the total distance among data points within the clusters of cellular expression data (within cluster dispersion) were plotted for cluster sizes from 10 to 65 over 100 simulations (Table S14) using Euclidean distance measure (De Hoon et al. 2004). Algorithms used for this analysis are available at http://bonsai.ims.u-tokyo.ac.jp/mdehoon/software/cluster/index.html. It was found that at approximately 45 clusters there was a pronounced discontinuity in the rate of change in the distance among points within the cluster, and this was adopted as a tentative minimal number of clusters.

#### Determination of cell-enriched expression in adult retina and retinal progenitor cells

For the data presented in Figure 5A, numerical cellular expression data from Table S7 was used. Transcripts were assayed as enriched in a specific cell type if they showed highest (but not necessarily exclusive) expression in the cell type in question after the first postnatal week of life. Genes enriched in subsets of bipolars or amacrines were treated as bipolar- and amacrine-enriched, respectively.

Whether or not a gene showed retinal-progenitor-enriched expression was determined from Table S7 by the following empirical set of criteria, which were found to cover virtually all known retinal-progenitor-enriched genes: early vO/svO or scO/sscO greater than 1, early (scO + sscO + vO + svO) greater than early (scI + sscI + vI + svI), early (vO + svO) greater than or equal to early (scO + sscO), and mid (vO + svO) greater than mid (scO + sscO). (See legend of Table S5 for a key to these abbreviations.)

To determine whether genes that are cell type–specific in the adult retina are disproportionately enriched in retinal progenitors (see Figure 5A), we have used the hypergeometric distribution statistical analysis to compute the probability that a subset of genes of a given size will have a given number of occurrences of the pattern we examine, when chosen randomly from the group of all known genes (Johnson et al. 1992).

#### Cluster analysis of SAGE data

Considering the numerous types of transcripts present in a cell or tissue and the small probability of sampling a particular type of transcript at each draw, the number of sampled transcripts of each type is assumed to be approximately Poisson distributed. Statistically, when this actual sampling process is random enough, Poisson would be the most practical and reasonable assumption compared to other probability models. This assumption, with the assumption that each tag is uniquely mapped to a transcript, leads to the probability model used for clustering analysis of SAGE data (below).

First, all SAGE tags were assigned at random to *k* groups. Second, a cluster center, which led to the expected expression pattern of each tag, was calculated for each cluster. Chi-square test statistics were used to measure the distance between the observed expression pattern and the expected expression pattern of a tag in a cluster. Third, using an iterative method, tags were moved between clusters, and intra- and intercluster distances were measured with each move. Tags were allowed to remain in the new cluster only if they were closer to it than to their previous cluster. Fourth, after each move, the expression vectors for each cluster were recalculated. Last, the shuffling proceeded until moving any more tags made the clusters more variable, increasing intracluster distances and decreasing intercluster dissimilarity (see Protocol S1 for full details of the algorithms used, as well as Cai, et al. 2004 for a more detailed discussion of applications of the protocol).

To compute optimal values for the number of clusters *k,* the within-cluster dispersion was computed for increasing values of *k*. This within-cluster dispersion declined as new clusters were added. We thus looked for the reduction at each step, and observed the rate of change. Discontinuities in the rate of change were taken to indicate that a meaningful cluster number had been obtained, with the lowest number of clusters that showed such a discontinuity being used for analysis (Hartigan 1975; Yeung et al. 2001).

In order to determine the optimal number of clusters to use in the analysis of the SAGE data, the within-cluster dispersion was determined for a range of ten to 65 clusters over 100 iterations. If certain numbers of clusters gave a better fit to the data, they should show discontinuities in the rate of decrease (Hartigan 1975). It was found that setting the number of *k*-means clusters at around 25, 40, and 55 showed these features (see Table S15)

#### Database construction

Data from 21 SAGE libraries and ISH images were gathered and stored in a MySQL relational database (http://www.mysql.com). Information on the measurement values for the SAGE libraries and ISH images can be accessed at http://134.174.53.82/cepko/. The database was developed to provide up-to-date mapping of SAGE tags to UniGene clusters. Since a single sequence tag can represent different genes and, conversely, an individual UniGene cluster can be represented by more than one tag, both “full” and “reliable” tag-to-UniGene mappings (Lash et al. 2000) have been created and can be selected by the user. The cluster assignments and their reliability were obtained from NCBI SAGEmap (http://www.ncbi.nlm.nih.gov/SAGE). For the database reported herein, UniGene Build 131 of Mus musculus and Build 164 of Homo sapiens (http://www.ncbi.nlm.nih.gov/UniGene) were used for the mappings. However, the database at http://134.174.53.82/cepko/ includes up-to-date mapping data. For each UniGene cluster, all measurement values and ISH images of associated tags are provided. Measurement values can also be segregated and summed up for each library if more than one SAGE tag is mapped to a given UniGene cluster. A plot of measurement values was also created to visualize patterns across the SAGE libraries. Additionally, for each UniGene cluster, links to gene functions using GO, accession numbers for annotated human orthologs, and LocusLink IDs have been provided.

## Supporting Information

Figures S2–S12 show ISH data for genes that show dynamic expression in developing retina. All pictures were obtained from central retina. Cellular laminae of both the developing and mature retina are indicated with colored bars. All pictures were taken at 200x. See Table S5 for a full list of probes used.

Figure S1 Comparison of E14.5 EST Versus E14.5 SAGE Data The number of times a gene was observed in a set of 15,268 individual ESTs obtained from E14.5 mouse retina (data obtained from Mu et al. [2001]) compared to a set of 15,268 individual E14.5 retinal SAGE tags generated in this study. Only genes present at least ten times in the EST data set were considered.
(1.7 MB TIF).

Figure S2 Heterogeneous Developmental Onset of Phototransduction Gene Expression The genes shown are *rod arrestin, PrCdh, Gγ1, rod PDEγ, rhodopsin, peripherin 2, Gα1,* and *GCAP1*.
(26.9 MB TIF).

Figure S3 Genes Expressed in Subsets of Cells in Developing ONBL Sections were from central retina. The genes shown are *Otx2, RORβ, Yboxbp1, Mm.38347, Mm.11660, BTF3, H2Ax, Ppp1r14b, Grb10, Mm.158631, HMG-AT1, Mm.24141, KIAA1411, Mm.25018, IAP5,* and *Chaf1b*.
(25.7 MB TIF).

Figure S4 Genes Expressed Broadly in Mitotic Progenitors The genes shown are *PDK3, Giα2, β-catenin, LRC8, Nrarp, Foxn4,* and *HMG-17*.
(27.4 MB TIF).

Figure S5 Genes Expressed in Undefined Subsets of Progenitors/Precursors The genes shown are *FABP7, BMP7, NTT7, Inhibin βB,* and *Sal3.*
(20.8 MB TIF).

Figure S6 Known Transcription Factors Expressed in Developing Rods These data are shown to allow direct comparison with the data in Figures 4 and S7. The genes shown are *NeuroD1, Crx, Nrl,* and *NR2E3.*
(24.9 MB TIF).

Figure S7 Genes Expressed in Developing Rods The genes shown are *Cpx2, TRABID, Fln29, Mak, Mm.24642, Nlk, Hrs, Tnfsf13,* and *Arip2.*
(18.3 MB TIF).

Figure S8 Genes Expressed in Developing Bipolar Cells The genes shown are *Chx10, Gli5, Dbp, Lhx4, Mm.41284, Prkcl, SEZ-6,* and *Zfh4.*
(21.4 MB TIF).

Figure S9 Genes Expressed in Developing Horizontal Cells The gene shown is *Borg4.*
(11.0 MB TIF).

Figure S10 Genes Expressed in Developing Amacrine Cells The genes shown are *Unc-51-like-1, ArfGAP, robo3, necdin, SAK, Mm.6393, Mm.34130, Nhlh2, NPY, Mm.21657, Mm.215653,* and *Mm.41638*.
(19.1 MB TIF).

Figure S11 Genes Expressed in Developing Müller Glia The genes shown are *KIAA0937, Mm.157502, Slc38a3, Nkd1, Dsp8, carbonic anhydrase 2,* and *cyclin D1.*
(40.1 MB TIF).

Figure S12 Additional Noncoding RNAs Expressed in Developing Retina The genes shown are *MEG3, Xist,* and *Tsix.*
(13.6 MB TIF).

Protocol S1 Description of Methodology Used for Cluster Analysis of SAGE Tags (52 KB DOC).

Table S1 Summary of SAGE Tag Distribution The total cumulative number of tags found at each abundance level in all 12 retinal libraries (i.e., the ten libraries from total retinal of wild-type animals, the library from P10.5 *crx^−/−^* animals, and the library from microdissected ONL of adult animals) is shown. The number of tags, and the fraction of total tags, that do not show any reliable match for any gene (data from NCBI) are also shown.
(14 KB XLS).

Table S2 Full List of Tag Counts in All SAGE Libraries Considered This list includes not only all libraries made from retinal tissue, but also nonretinal SAGE libraries made by this group, and other mouse libraries that are publicly available. Raw, unnormalized tag counts are shown. See Materials and Methods for more details on the SAGE libraries analyzed.
(17.9 MB TXT).

Table S3 Twenty-Four-Cluster Analysis for SAGE Tags All tag abundance levels were normalized to 100,000. Tags present at greater than 0.1% in one or more of the ten wild-type total retina libraries were considered. The single most probable “reliable” tag-to-gene match (http://www.ncbi.nlm.nih.gov/SAGE) is shown, along with the confidence level of that assignment. Mouse UniGene number is shown for each tag-to-gene match, along with LocusLink ID, where available. In each case where a gene was analyzed by ISH in developing retina, that fact is indicated in the final column. In some cases, a gene that matched the tag with a lower confidence level was tested. In these cases, the UniGene number of the gene tested by ISH differs from that of the most probable tag match.
(1.0 MB XLS).

Table S4 Molecular Function, Biological Process, and Subcellular Compartment GO Data Are Shown for Each Gene Analyzed by ISH in the Retina Gene names and LocusLink IDs for these genes are also shown
(225 KB XLS).

Table S5 Complete List of Cellular Expression Patterns for Each Probe Tested The SAGE tag matching each gene tested is given, as well as the accession number of the cDNA used to generate each probe used for ISH. Cellular expression is scored on a 0–5 point scale for each time point tested, as well as for E16 embryo and P6 head cut in horizontal section. A, amacrine cells; Ast, astrocytes; B, bipolar cells; Bv, blood vessels; Cb, cerebellum; CM, ciliary margin; CP, cortical plate; Ctx, cerebral cortex; DG, dentate gyrus of hippocampus; DRG, dorsal root ganglia; EGL, external granule layer of developing cerebellum; EOM, extraocular muscles; G, ganglion cells; H, horizontal cells; Hippo, hippocampus; I, inner neuroblastic layer; In, inner nuclear layer; MG, Müller glia; MGE, medial ganglionic eminence; ND, not determined; O, outer neuroblastic layer; OB, olfactory bulb; OE, olfactory epithelium; ORN, olfactory receptor neurons; P, panretinal; PC, Purkinje cells; PNS, peripheral nervous system; Pr, photoreceptors; Pr(is), inner segments of photoreceptors; sA, subset of amacrine cells; sB, subset of bipolar cells; SC, spinal cord; sG, subset of ganglion cells; sI, subset of cells in INBL; sIn, subset of cells in INL; scI, scleral INBL; sscI, subset of cells in scleral INBL; svI, subset of cells in vitreal INBL; sO, subset of cells in outer neuroblastic layer; scO, scleral ONBL; sscO, subsets of cells in scleral ONBL; svO, subset of cells in vitreal ONBL; sPr, subset of photoreceptors; SVZ, subventricular zone; vI, vitreal INBL; vO, vitreal ONBL; VRN, vomeronasal receptor neurons; VZ, ventricular zone.
(381 KB XLS).

Table S6 User-Curated Cellular Expression Clusters for Genes Tested by ISH in Retina Here, data from Table S5 are summarized such that the predominant cellular expression pattern from early (E12–E18), mid (P0–P4), and late (P6–adult) developing retina is recorded, and genes are grouped into coexpressed clusters by user annotation. The main cell types expressing the gene in the retina over the interval in question are listed, with weaker expression in other cell types being noted in parentheses. Clusters are given a name (after a representative gene) and a unique cluster number, and the presumptive cell types that show greatest expression are listed. Genes for which the full developmental expression profile was not determined are tentatively assigned to clusters that showed the best fit based on two out of three criteria, with tentative assignments being indicated as such
(261 KB XLS).

Table S7 Numerical Cellular Expression Data Used for Machine-Aided Cluster Analysis of Cellular Expression Patterns of Genes Tested by ISH in Retina To obtain these numbers, data from Table S5 were modified. As in Figure S6, expression data were summarized for early (E12–E18), mid (P0–P4), and late (P6–adult) developing retina. In cases where cellular expression changed dramatically within one of these three intervals (e.g., expression shifted from INBL to ONBL), these cellular expressions were both entered in the category in question. Genes that were not examined in all three of these time intervals were not considered in this analysis. Cellular expression data, scored on a 0–5 point scale, were then entered for each time point separately in each of the categories used to score retinal cellular expression in Table S5.
(266 KB XLS).

Table S8 Comparison of User-Curated Cellular Expression Clusters from Table S6 and a 45-Cluster Machine-Aided Analysis of the Cellular Expression Data from Table S7
The fraction listed notes the fraction of genes in the machine-generated cluster that were found in a given user-curated cellular expression cluster. The presumptive cellular expression pattern of each user-curated cellular expression cluster is also listed (following Table S6).
(86 KB XLS).

Table S9 Comparison of 4N-Enriched Genes from Livesey et al. (2004) and SAGE Cluster Data from Table S3
Shown is the percentage of tags that matched genes enriched in 4N retinal progenitor cells found in a given SAGE tag cluster.
(14 KB XLS).

Table S10 Comparison of the SAGE Tag Cluster Data from Table S3 and the 72-Cluster Analysis of the User-Curated Cellular Expression Data from Table S6
Values indicate the fraction of all tags found in a given SAGE tag cluster that were found in a specific user-curated cellular expression cluster. The presumptive cellular expression pattern of each cellular expression cluster is also listed (following Table S6).
(209 KB XLS).

Table S11 SAGE Tags Representing the Known Photoreceptor-Specific Genes Analyzed in Figure S2
Tags in each library are expressed as the fraction of all tags that match the gene in question that were found in the ten libraries considered.
(15 KB XLS).

Table S12 Candidate Noncoding RNAs Analyzed by ISH in This Study The SAGE tag corresponding to the transcript in question is listed, along with UniGene numbers, and accession numbers of the probes used for ISH for each candidate noncoding RNA. *P*-values for BLASTN and BLASTX mouse/human comparisons are shown. Transcripts that show high BLASTN, but low BLASTX, matches to human may represent the best candidates for noncoding mRNAs of functional importance and are indicated as likely to be genuine noncoding RNAs. NS, not significant.
(17 KB XLS).

Table S13 Accession Numbers for Full-Length Transcripts for Genes Tested by ISH in This Study, Along with Their Human Orthologs Chromosomal localizations are shown for both the mouse genes and their human orthologs. Genes located within chromosomal intervals containing mapped but uncloned retinal disease genes are indicated by the name of the disease (terminology from Retnet; http://www.sph.uth.tmc.edu/Retnet/disease.htm). User-curated cellular expression data of the genes in question (derived from Table S6) are shown to aid in prioritizing candidate disease genes for further investigation. ND, not determined.
(291 KB XLS).

Table S14 Average Distance Analysis of Cellular Expression Data from Table S7
The values shown here are the average sum-of-squares within *k-*means clusters over all variables. Euclidian mean distance–directed clustering is used (Hartigan 1975). The proportional reduction of error (PRE) for each number of clusters is also shown. This measures the ratio of reduction in within-cluster dispersion to the previous within-cluster dispersion (Hartigan 1975). For this analysis, PRE is given by (*Ni* − *N*(*i* – 5))/*Ni,* where *N* is the average within-cluster distance and *i* is cluster number.
(14 KB XLS).

Table S15 Average Distance Analysis of SAGE Tag Clusters Tags present at greater than 0.1% in one or more of the ten wild-type total retina libraries were considered and were normalized to 100,000 for this analysis. The average sum-of-squares within *k-*means clusters for each number of clusters is shown. The PRE, given by (*Ni* − *N*(*i* – 5))/*Ni,* is also shown.
(14 KB XLS).

### Accession Numbers

The GenBank (www.ncbi.nlm.nih.gov) accession numbers for the genes discussed in this paper are β-catenin (NM_007614), *ArfGAP* (BC030682), *Arip2* (NM_025292), *BMP7* (NM_007557), *Borg4* (NM_012121), *brain fatty acid binding protein 7* (NM_021272), *BTF3* (NM_145455), *carbonic anhydrase 2* (NM_009801), *cdk4* (NM_009870), *Chaf1b* (NM_028083), *Chx10* (NM_007701), *Cpx2* (NM_007756), *Crx* (NM_007770), *Dbp* (NM_016974), Drosophila castor gene (BC035954), *Dsp8* (XM_181424), *FABP7* (NM_021272), *Fln29* (NM_172275), *Foxn4* (NM_148935), *GCAP1* (NM_008189), *Giα2* (NM_008138), *Gli5* (NM_031184), *Grb10* (NM_010345), *Gα1* (NM_008140), *Gγ1* (NM_010314), *H2Ax* (NM_010436), *HMG-17* (NM_016957), *HMG-AT1* (NM_016660), *Hrs* (NM_008244), *IAP5* (NM_009689), *inhibin βB* (BC048845), *KIAA0937* (NM_172442), *KIAA1411* (NM_026604), *Lhx4* (NM_010712), *LRC8* (NM_172736), *Mak* (NM_008547), *MEG3* (NM_144513), *Mm.103742/Cdc42GAP* (NM_020260), *Mm.11660* (AK034313)*, Mm.11738/Ark-1* (BC005425)*, Mm.142856/Lhx2* (NM_010710), *Mm.150838/RNCR1* (AK044330), *Mm.157502* (NM_026592)*, Mm.158631* (XM_132295)*, Mm.1635/PIAS3* (NM_018812), *Mm.18789/Sox4* (NM_009238), *Mm.19155/sFrp2* (NM_009144), *Mm.193526/Yboxbp4* (NM_007705), *Mm.194050/RNCR3* (AK044422), *Mm.200608/clusterin* (NM_013492), *Mm.20465/GPCR37* (NM_010338), *Mm.213213/HK-R* (NM_145419), *Mm.215653* (NM_183191), *Mm.21657* (BC038057), *Mm.2214/septin 4* (NM_011129), *Mm.22288/cyclin D1* (NM_007631), *Mm.2229/Eya2* (NM_010165)*, Mm.235550/ERRβ* (NM_011934), *Mm.23916* (AK009781), *Mm.24141* (NM_025615), *Mm.24642* (NM_146168), *Mm.25018* (BC010304), *Mm.26062/AD024* (NM_025565), *Mm.27953/glycine decarboxylase* (NM_138595), *Mm.29067/Mbtd1* (NM_134012), *Mm.29496/Zf-1* (AK004085), *Mm.29729/Tweety1* (NM_021324), *Mm.29924/Arl6ip1* (BC010196)*, Mm.34130* (AK012601), *Mm.34701/Pum1* (NM_030722), *Mm.3499/Rax homeodomain factor* (NM_013833), *Mm.35817* (NM_145940), *Mm.35829/Edr* (NM_133362), *Mm.38347* (XM_126644), *Mm.3904/Fgf15* (NM_008003), *Mm.40321/Pgrmc2* (XM_130859), *Mm.41284* (NM_153137), *Mm.41638* (NM_029530), *Mm.44854/RNCR2* (AK028326), *Mm.4541/Sox2* (NM_011443), *Mm.45753/KIAA0013* (NM_181416), *Mm.4605/Tbx2* (NM_009324), *Mm.5021/DDR1* (NM_007584), *Mm.55143/Dkk3* (NM_015814), *Mm.6393* (NM_010045), *Mm.89623/mCas* ( BC035954)*, Mm.9114/mu-crystallin* (NM_016669), *necdin* (NM_010882), *NeuroD1* (NM_010894 , *Neuropeptide Y* (NM_023456), *Nhlh2* (NM_178777), *Nkd1* (NM_027280), *Nlk* (NM_008702), *NPY* (NM_023456), *NR2E3* (NM_013708), *Nrarp* (NM_025980), *Nrl* (NM_008736), *NTT7* (NM_175328), *Otx2* (NM_144841), *PDK3* (NM_005391), *peripherin 2* (NM_008938), *Ppp1r14b* (NM_008889), *PrCdh* (NM_130878), *Prkcl* (NM_008857), *RGPRIP* (NM_023879), *rhodopsin* (NM_145383), *robo3* (NM_011248), *rod arrestin* (NM_009118), *rod PDEγ* (NM_012065), *RORβ* (NM_146095), *SAK* (NM_019945), *Sal3* (NM_026528), *SEZ-6* (NM_021286), *Slc38a3* (NM_023805), *syntrophin-associated kinase* (NM_019945), *Tnfsf13* (NM_023517), *TRABID* (AK005926), *Tsix* (AF138745), *Unc-51-like-1* (NM_009469), *Xist* (AK011511), *Yboxbp1* (NM_011732), and *Zfh4* (NM_030708).

## Abbreviations

CNS: central nervous system
E[number]: embryonic day [number]
EST: expressed sequence tag
GCL: ganglion cell layer
GO: Gene Ontology Consortium
INL: inner nuclear layer
INBL: inner neuroblastic layer
ISH: in situ hybridization
NCBI: National Center for Biotechnology Information
ONL: outer nuclear layer
ONBL: outer neuroblastic layer
ORF: open reading frame
P[number]: postnatal day [number]
RNCR: retinal noncoding RNA
SAGE: serial analysis of gene expression
