# Supplementary figures and images for: Genomic Analysis of Mouse Retinal Development
Source: PLoS Biol. 2004 Jun 29;2(9):e247. doi: 10.1371/journal.pbio.0020247 (PMC439783; doi:10.1371/journal.pbio.0020247)

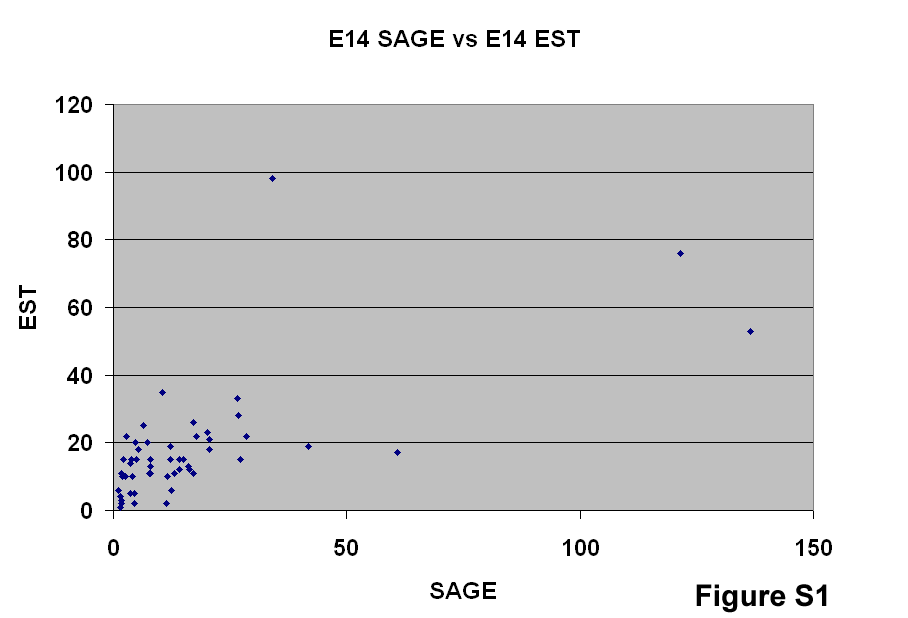

Supplement: Figure S1 — The number of times a gene was observed in a set of 15,268 individual ESTs obtained from E14.5 mouse retina (data obtained from Mu et al. [2001]) compared to a set of 15,268 individual E14.5 retinal SAGE tags generated in this study. Only genes present at least ten times in the EST data set were considered. (1.7 MB TIF). [file pbio.0020247.sg001.tif]

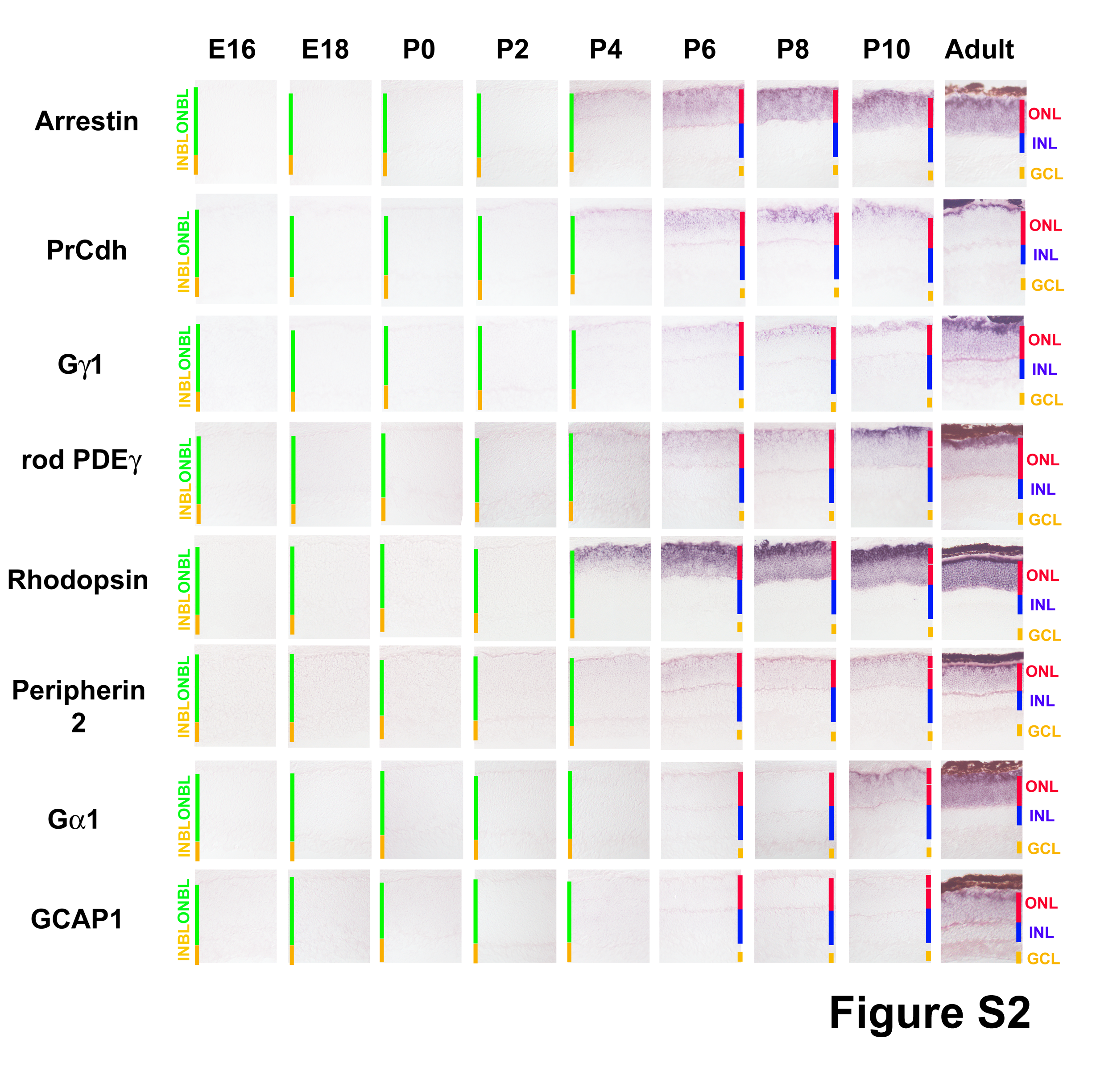

Supplement: Figure S2 — The genes shown are rod arrestin, PrCdh, Gγ1, rod PDEγ, rhodopsin, peripherin 2, Gα1, and GCAP1. (26.9 MB TIF). [file pbio.0020247.sg002.tif]

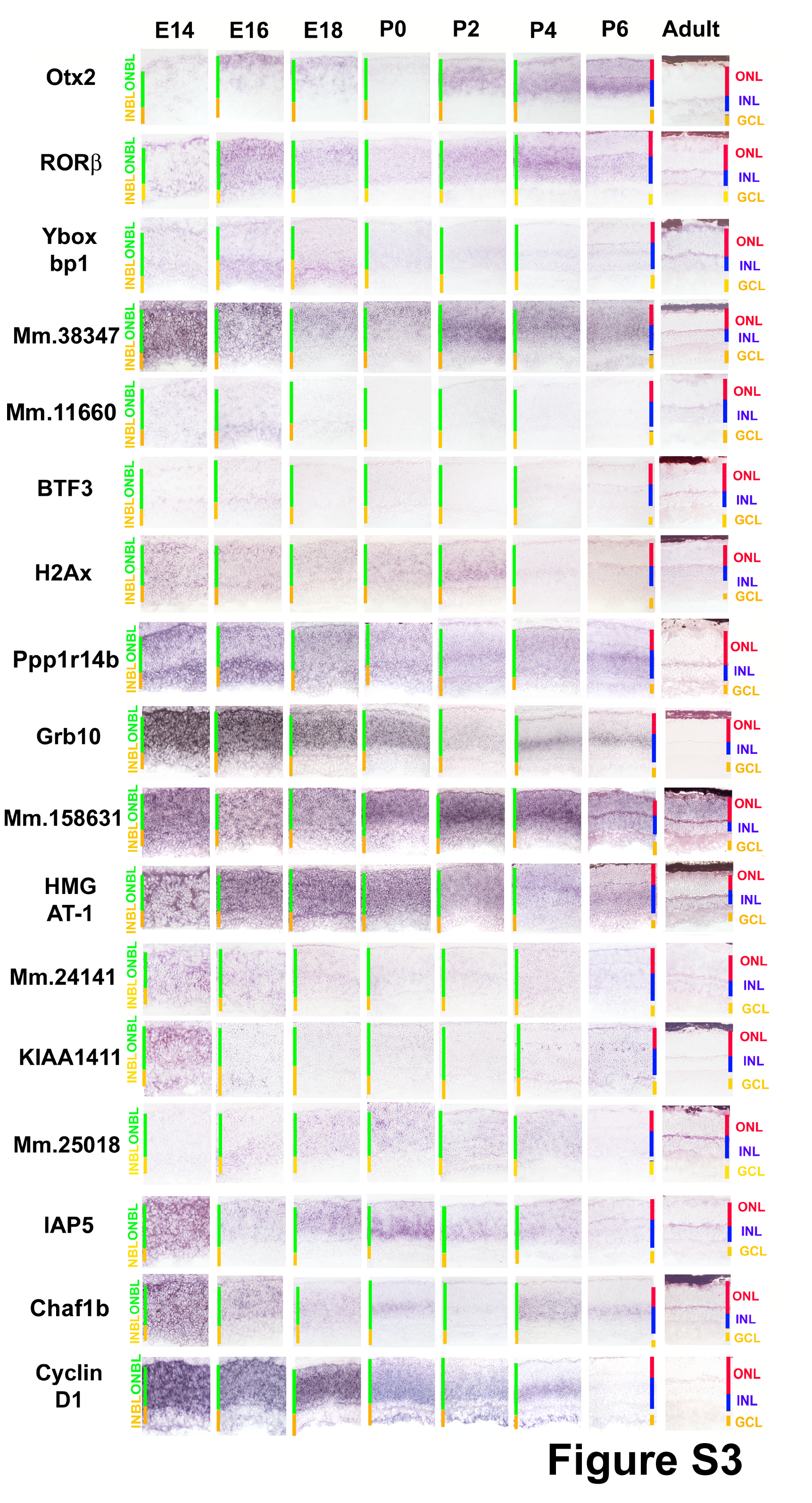

Supplement: Figure S3 — Sections were from central retina. The genes shown are Otx2, RORβ, Yboxbp1, Mm.38347, Mm.11660, BTF3, H2Ax, Ppp1r14b, Grb10, Mm.158631, HMG-AT1, Mm.24141, KIAA1411, Mm.25018, IAP5, and Chaf1b. (25.7 MB TIF). [file pbio.0020247.sg003.tif]

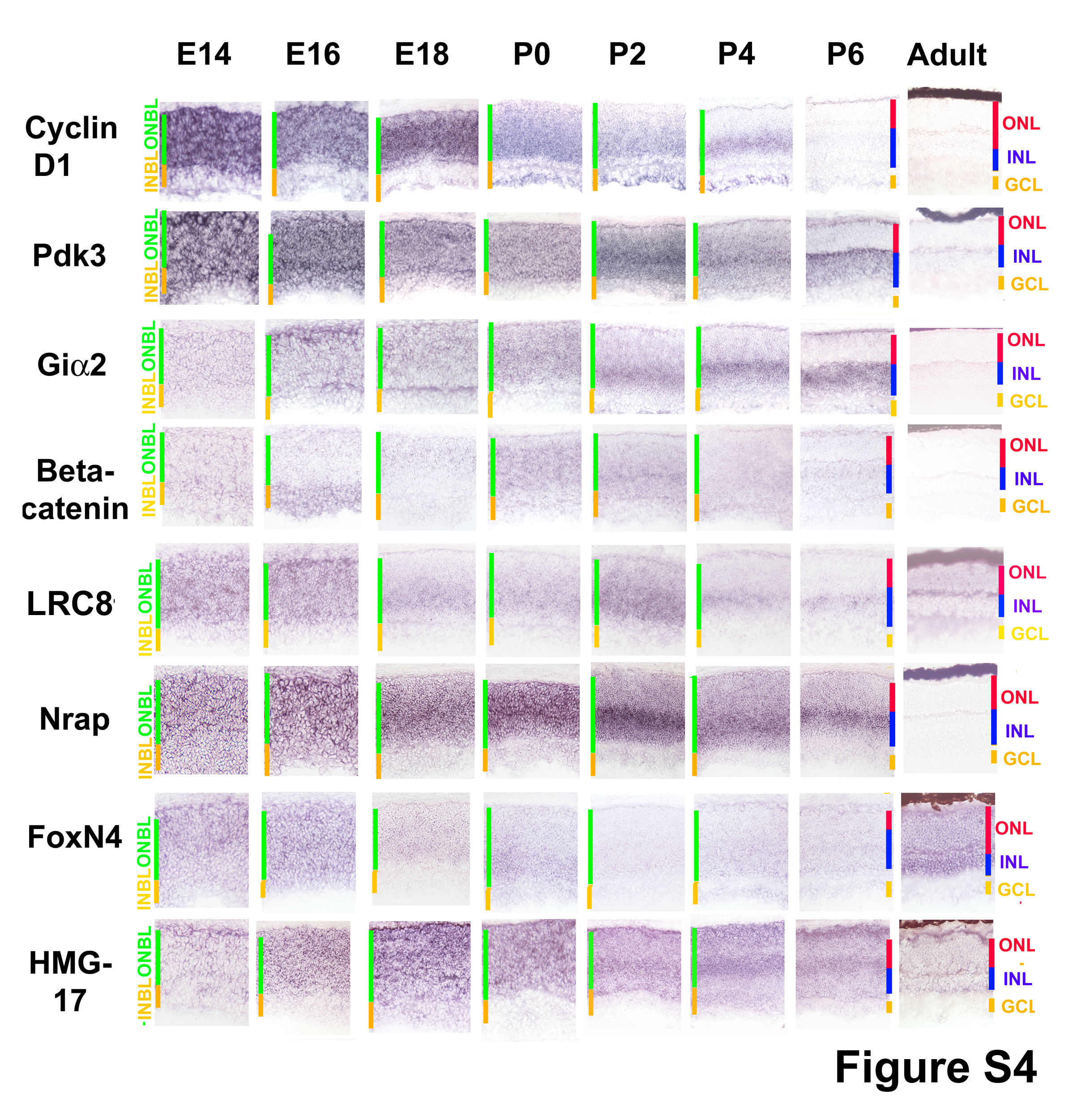

Supplement: Figure S4 — The genes shown are PDK3, Giα2, β-catenin, LRC8, Nrarp, Foxn4, and HMG-17. (27.4 MB TIF). [file pbio.0020247.sg004.tif]

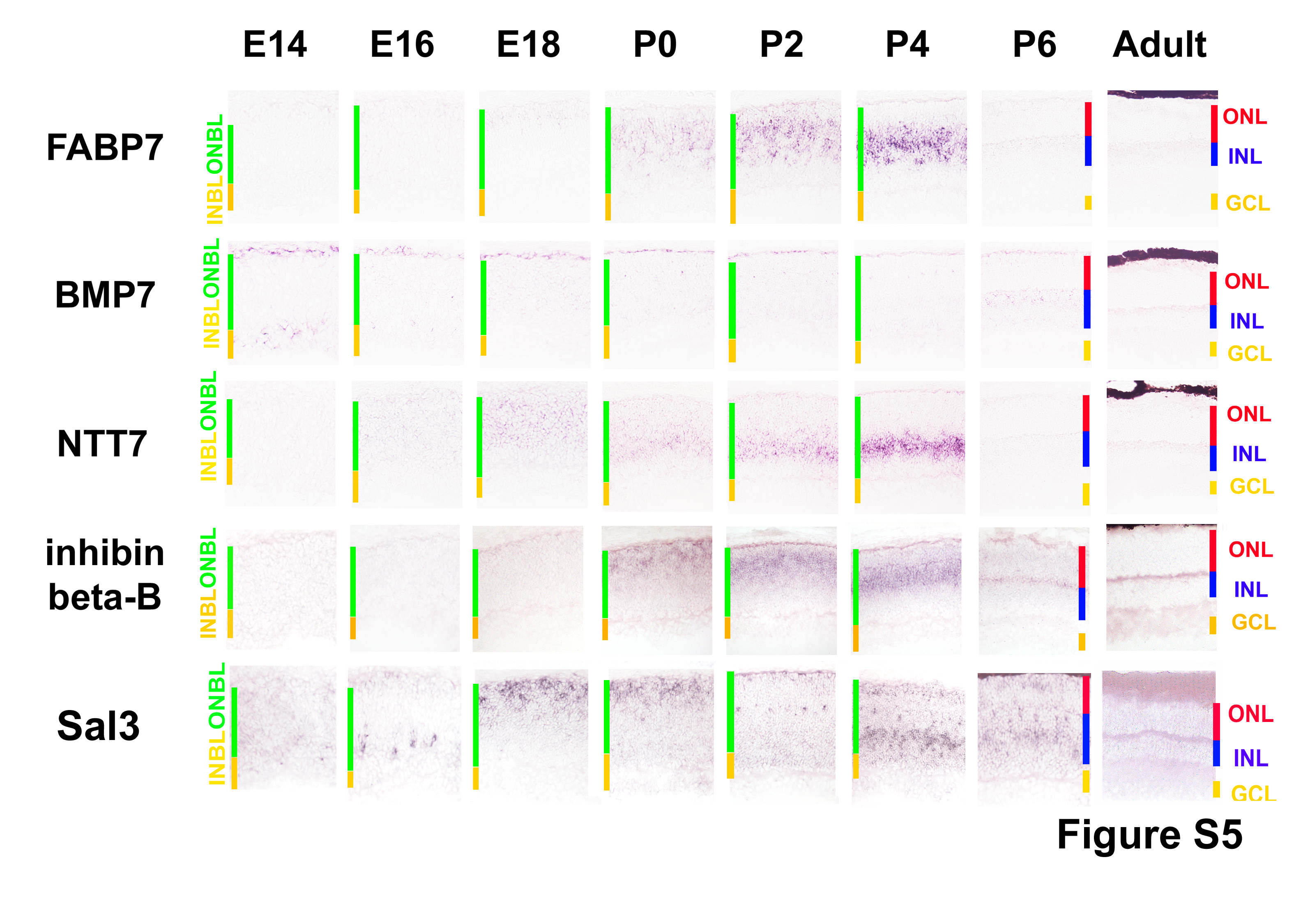

Supplement: Figure S5 — The genes shown are FABP7, BMP7, NTT7, Inhibin βB, and Sal3. (20.8 MB TIF). [file pbio.0020247.sg005.tif]

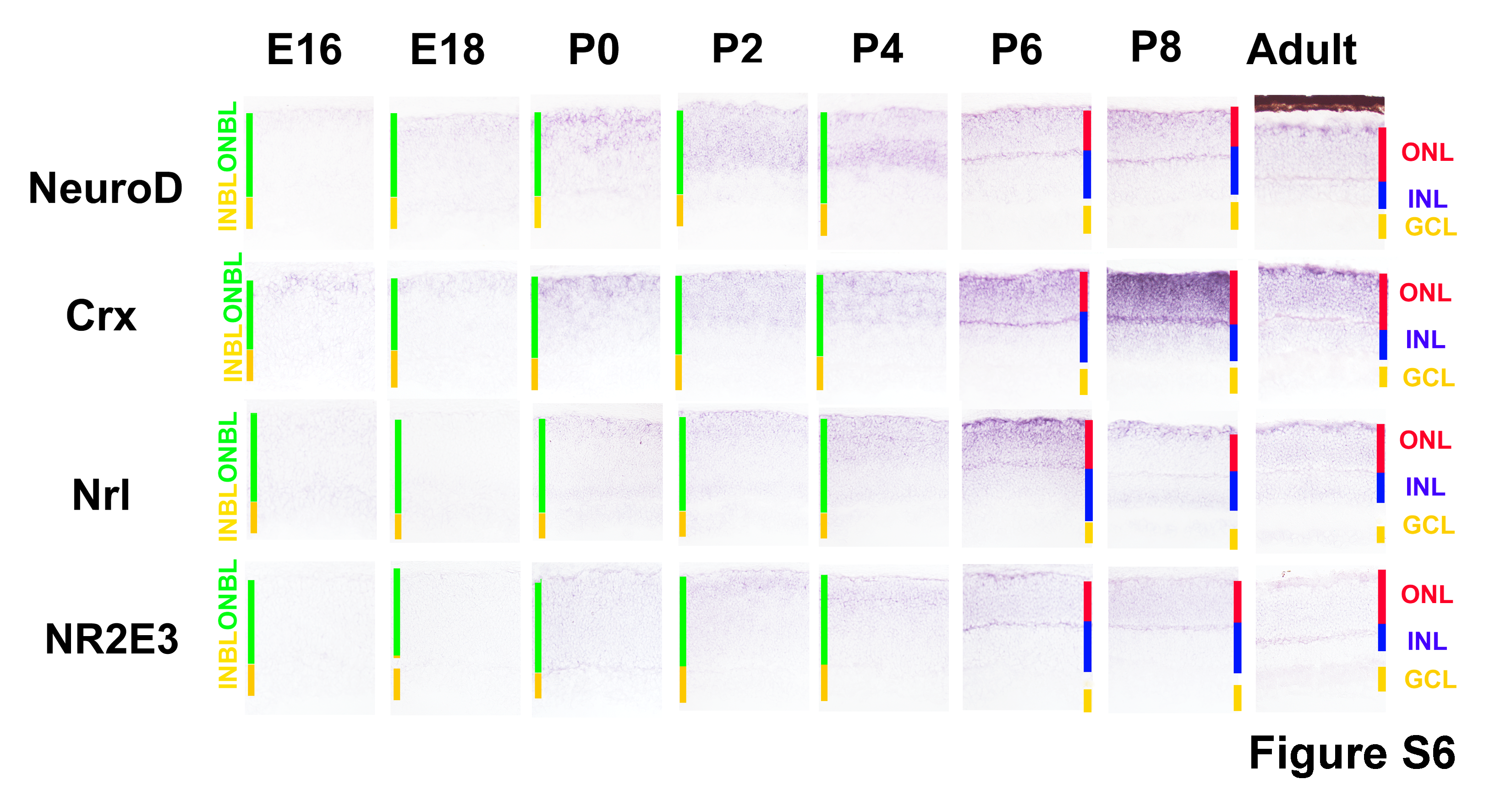

Supplement: Figure S6 — These data are shown to allow direct comparison with the data in Figures 4 and S7. The genes shown are NeuroD1, Crx, Nrl, and NR2E3. (24.9 MB TIF). [file pbio.0020247.sg006.tif]

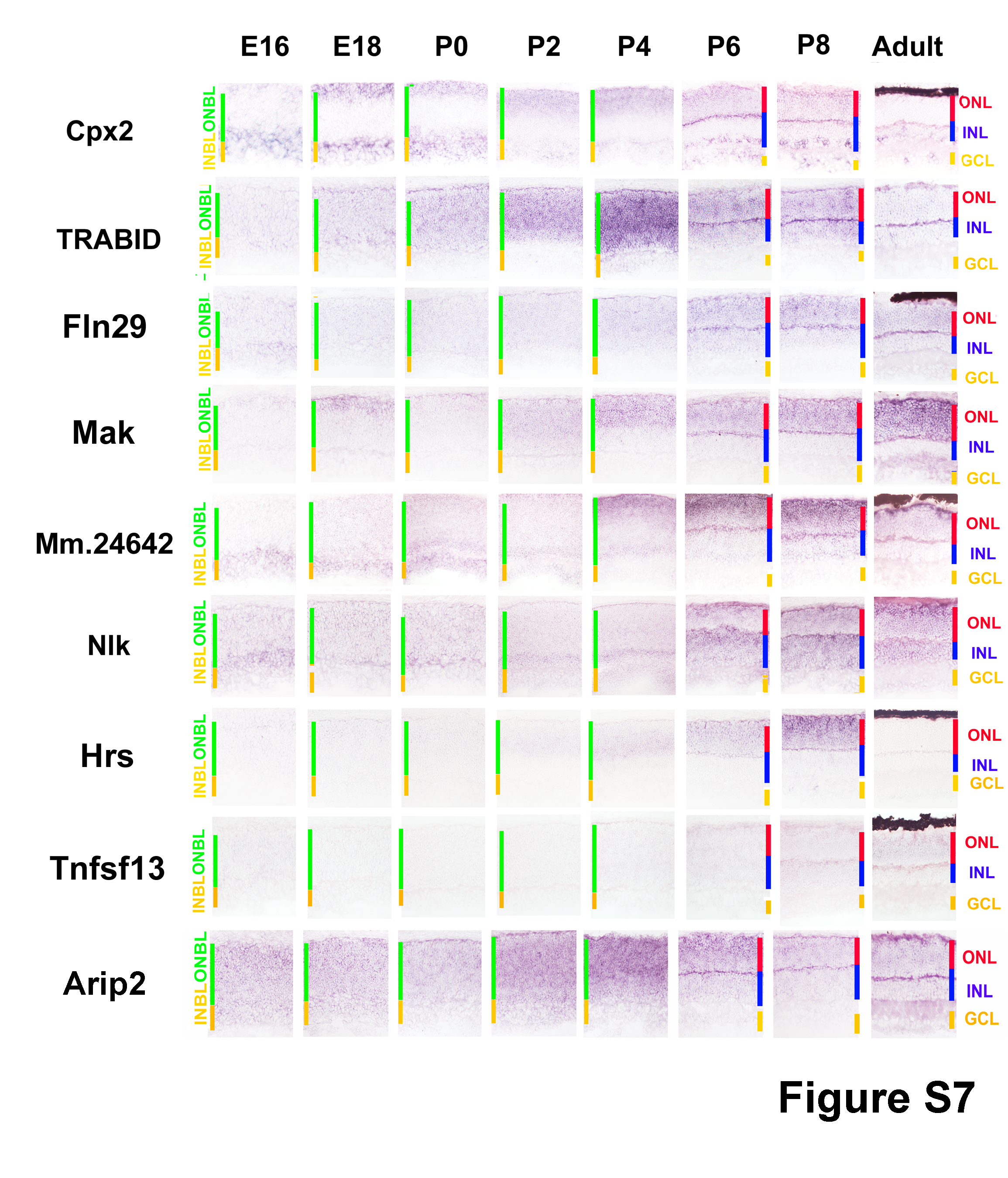

Supplement: Figure S7 — The genes shown are Cpx2, TRABID, Fln29, Mak, Mm.24642, Nlk, Hrs, Tnfsf13, and Arip2. (18.3 MB TIF). [file pbio.0020247.sg007.tif]

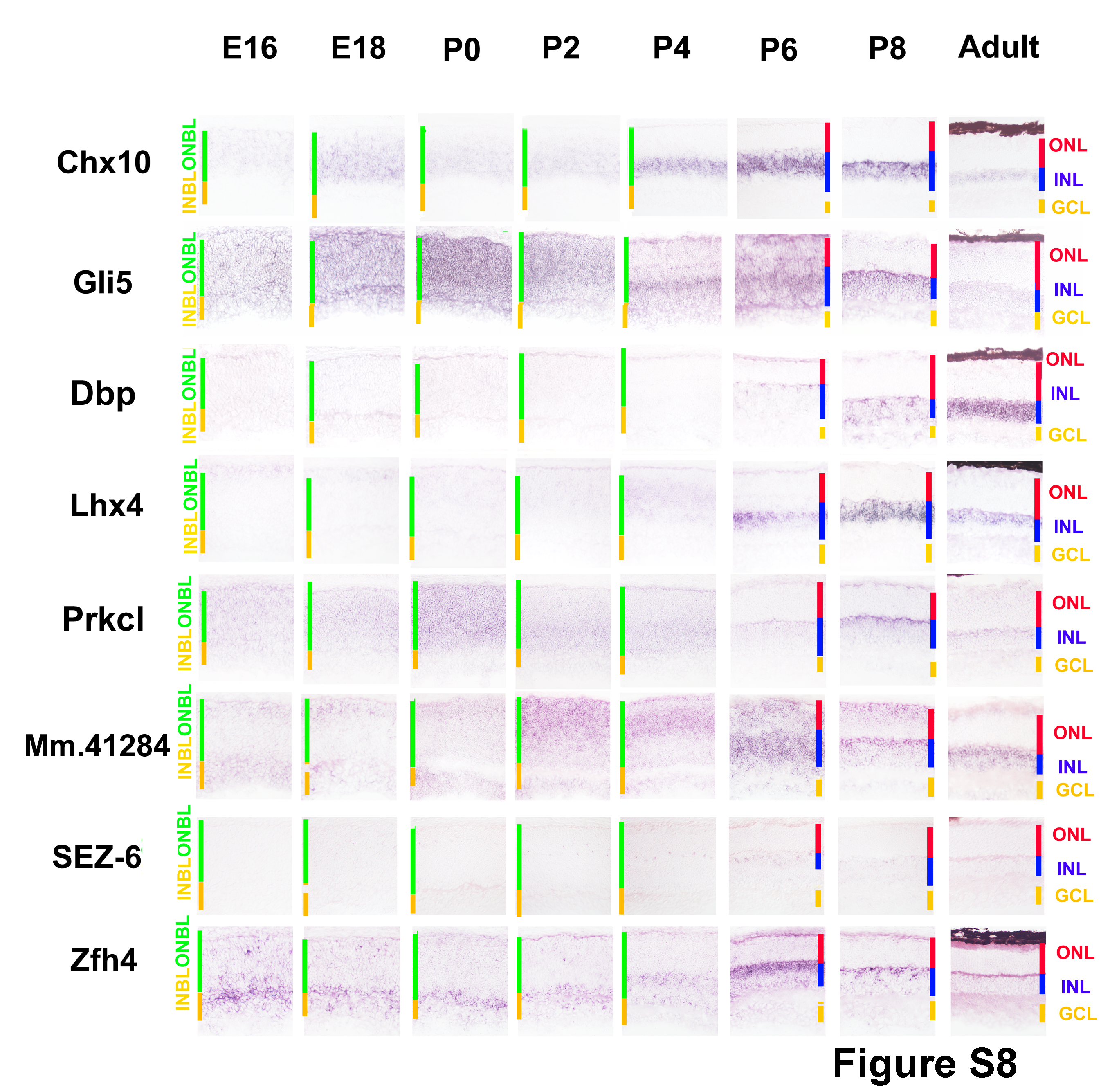

Supplement: Figure S8 — The genes shown are Chx10, Gli5, Dbp, Lhx4, Mm.41284, Prkcl, SEZ-6, and Zfh4. (21.4 MB TIF). [file pbio.0020247.sg008.tif]

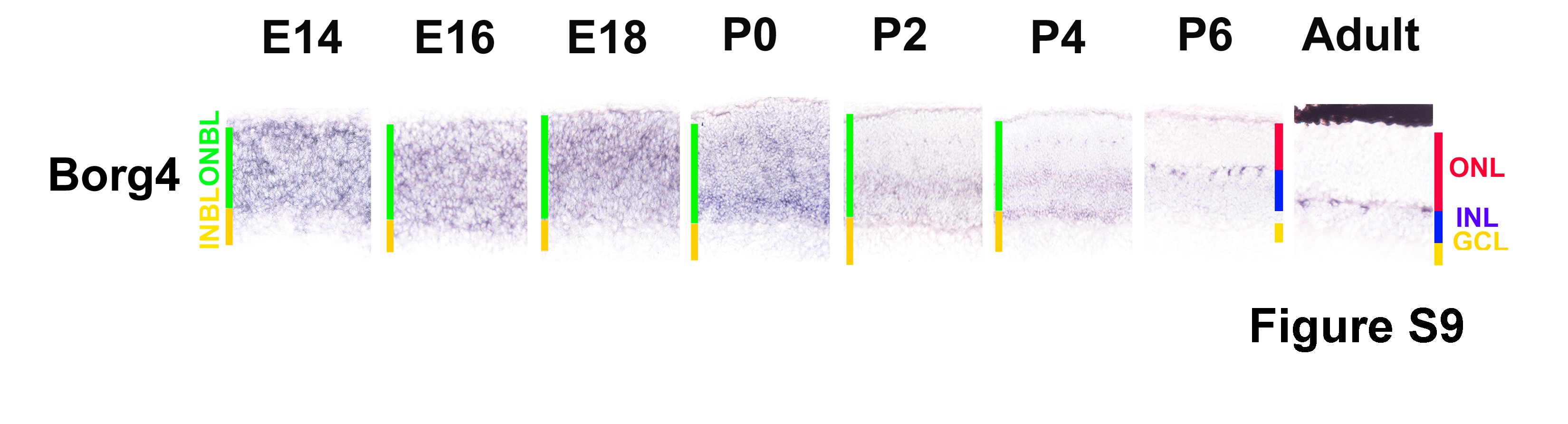

Supplement: Figure S9 — The gene shown is Borg4. (11.0 MB TIF). [file pbio.0020247.sg009.tif]

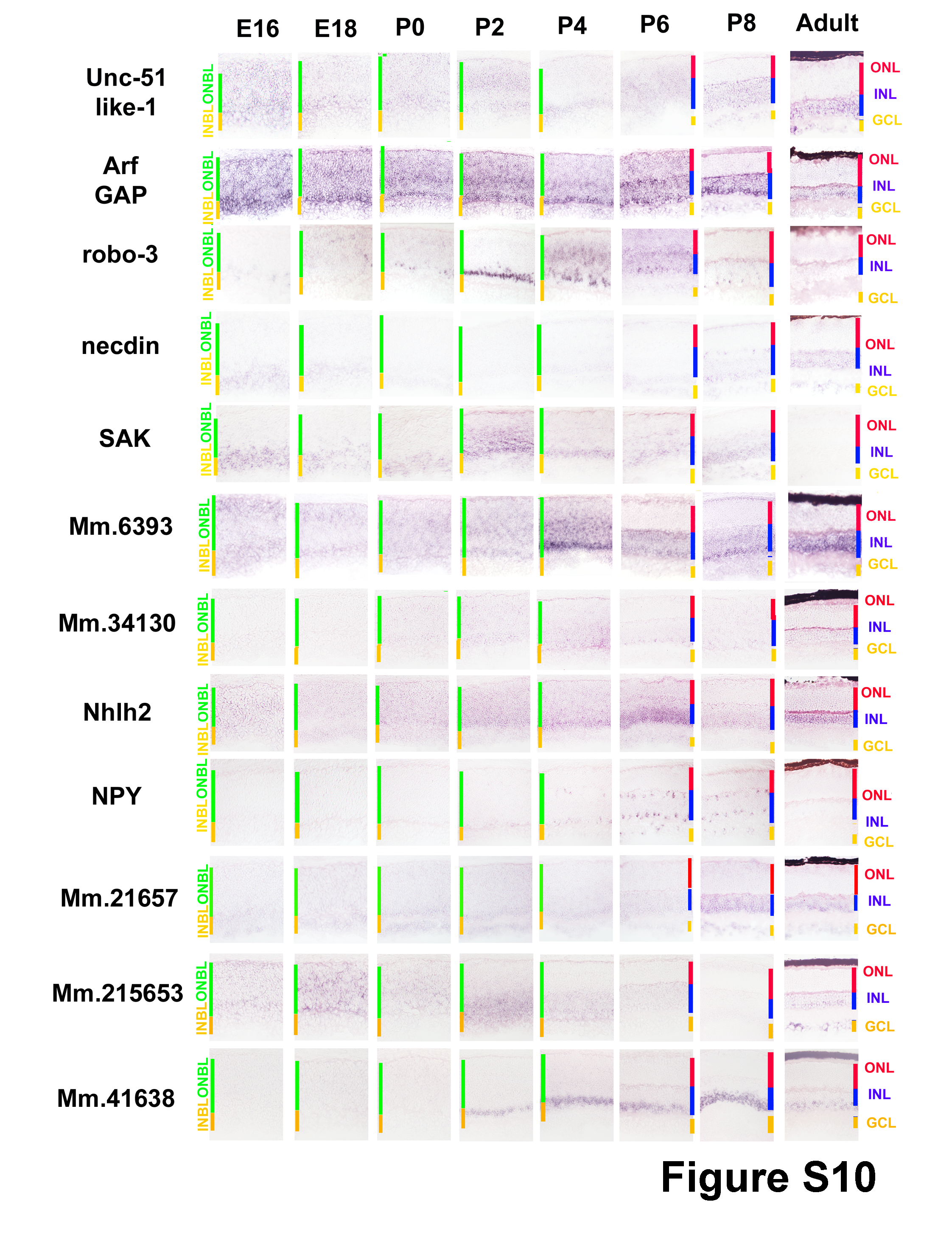

Supplement: Figure S10 — The genes shown are Unc-51-like-1, ArfGAP, robo3, necdin, SAK, Mm.6393, Mm.34130, Nhlh2, NPY, Mm.21657, Mm.215653, and Mm.41638. (19.1 MB TIF). [file pbio.0020247.sg010.tif]

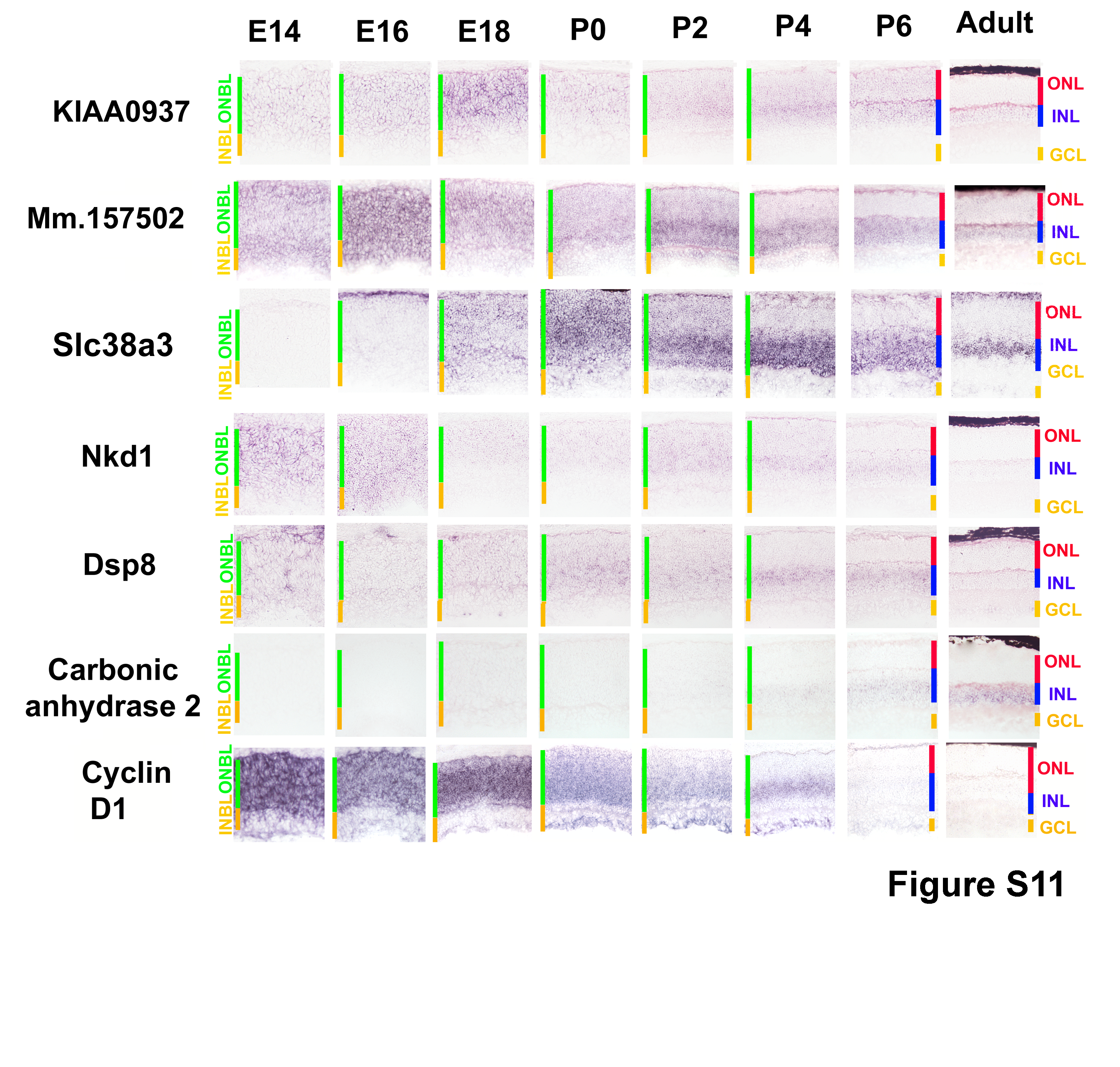

Supplement: Figure S11 — The genes shown are KIAA0937, Mm.157502, Slc38a3, Nkd1, Dsp8, carbonic anhydrase 2, and cyclin D1. (40.1 MB TIF). [file pbio.0020247.sg011.tif]

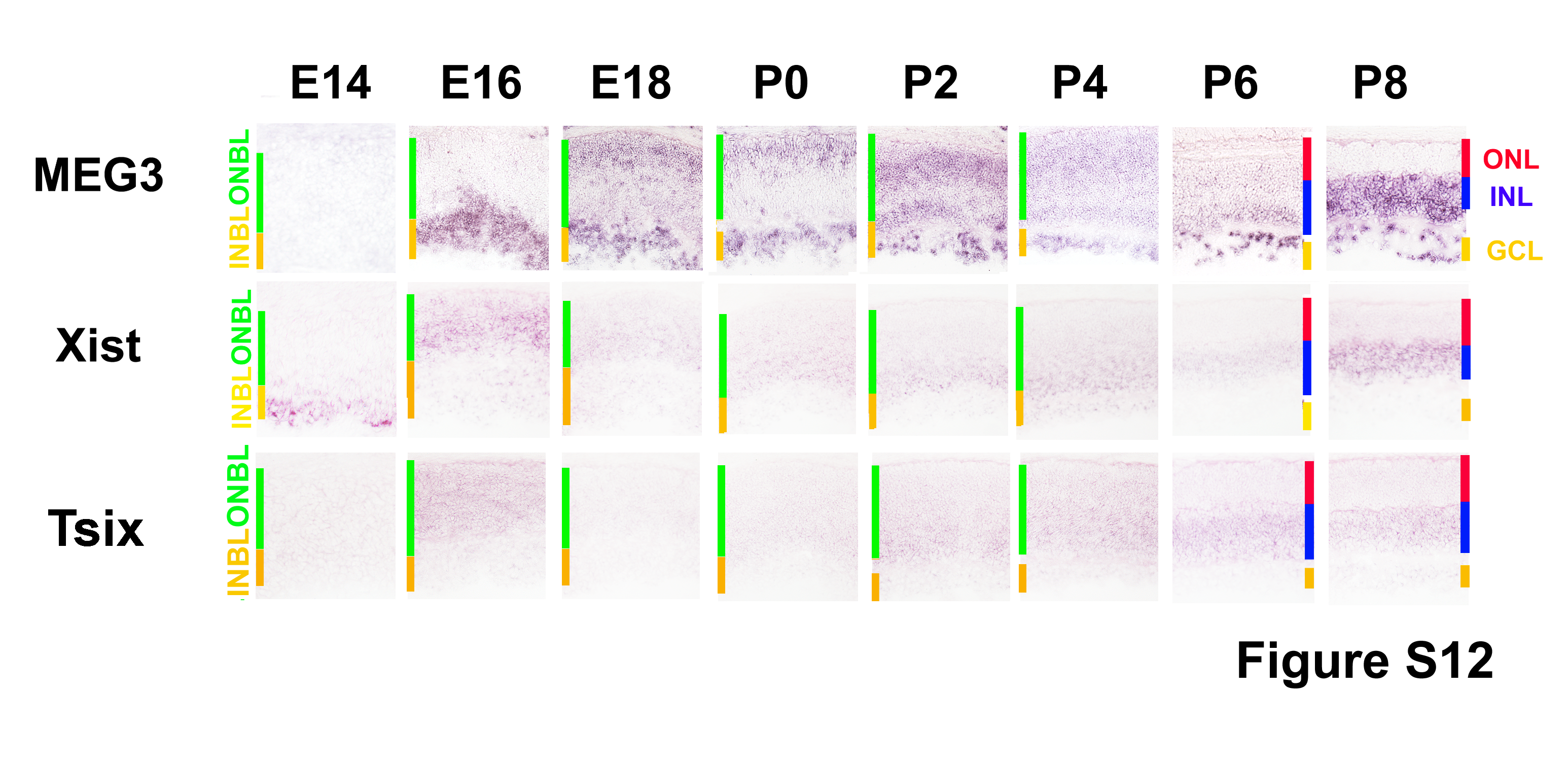

Supplement: Figure S12 — The genes shown are MEG3, Xist, and Tsix. (13.6 MB TIF). [file pbio.0020247.sg012.tif]
