# Supplementary material for: Genomic Analysis of Mouse Retinal Development
Source: PLoS Biol. 2004 Jun 29;2(9):e247. doi: 10.1371/journal.pbio.0020247 (PMC439783; doi:10.1371/journal.pbio.0020247)
Supplement: Protocol S1 — (52 KB DOC). [file pbio.0020247.sd001.doc]

**METHODS AND IMPLEMENTATION**

**Poisson assumption**

In a SAGE experiment, a set of transcripts from a cell will be sampled for tag extraction. Considering the numerous types of transcripts present in a cell and the small probability of sampling a particular type of transcript at each draw, we assume that the number of sampled transcripts of each type is approximately Poisson distributed. Statistically, when this actual sampling process is random enough, Poisson would be the most practical and reasonable assumption compared to other probability models. These arguments, with the assumption that each tag is uniquely mapped to a transcript, lead to our below probability model used for clustering analysis of SAGE data.

**Probability model**

We assume that the count for tags of same type in a SAGE library follows a Poisson distribution, and for different types of tags, their distributions are independent of each other.

Let *Y_i_(t)* be the count of tag *i* at condition *t*, and *Y_i_(t)~*Poisson(*λ_i_(t)θ_i_*). Here *λ_i_(t)θ_i_* denotes the expected count for tag *i* at condition *t* under the Poisson process. The expected count *λ_i_(t)θ_i_* consists of two factors: *θ_i_* and *λ_i_(t)*. *θ_i_* is the expected total count of transcript *i* (mapped to tag *i*) over all conditions; *λ_i_(t)* is the contribution of transcript *i* at condition *t* to the total count (*θ_i_*) expressed by percentage. Thus $\sum_{t=1}^{T} \lambda_{i}(t)=1$ when *T* conditions are considered.

The goal is to group together the transcripts with similar expression variation over different conditions; that is to cluster tags by their *λ_i_(t)*s. We thus assume that the tags within a cluster share the same ***λ=****(λ(1), λ(2), …, λ(T))*, where ***λ*** represents the cluster model. Further, based on the Poisson assumption and letting ***Y_i_****=(Y_i_(1), Y_i_(2), …, Y_i_(T))*, we have the following joint likelihood function for tags *i_1_, i_2_, …, i_m_* within a cluster:

$f(Y_{i_{1}},Y_{i_{2}},...,Y_{i_{m}}|\lambda,\theta_{i_{1}},\theta_{i_{2}},...,\theta_{i_{m}})=\prod_{k=1}^{m} \prod_{t=1}^{T} \frac{\exp(-\lambda(t)\theta_{i_{k}})(\lambda(t)\theta_{i_{k}})^{Y_{i_{k}}(t)}}{Y_{i_{k}}(t)!}$. (1)

The maximum likelihood estimators of *λ*s and *θ*s are:

$\widehat{\theta_{i_{k}}}=\sum_{t} Y_{i_{k}}(t)$, and $\widehat{\lambda}(t)=\frac{\sum_{k=1}^{m} Y_{i_{k}}(t)}{\sum_{k=1}^{m} \widehat{\theta_{i_{k}}}}=\frac{\sum_{k=1}^{m} Y_{i_{k}}(t)}{\sum_{k=1}^{m} \sum_{t} Y_{i_{k}}(t)}$. (2)

Thus for a set of tags assumed to be in the same cluster, we can estimate the cluster model ***λ*** and the total count *θ_i_* of each tag by (2). Further, we can use Chi-square test statistics to evaluate how well the observed tag count fits the estimated cluster model, which is to calculate S=$\sum_{k} \sum_{t} \frac{(Y_{i_{k}}(t)-\widehat{\lambda}(t)\widehat{\theta_{i_{k}}})^{2}}{\widehat{\lambda}(t)\widehat{\theta_{i_{k}}}}$. The bigger the value of S is, the less likely that the tags share same patterns of expression. Using Chi-square test statistics, the penalty for deviation from large expected counts is much smaller than that for small expected counts. This is consistent with the joint likelihood function $f(Y_{i_{1}},Y_{i_{2}},...,Y_{i_{m}}|\widehat{\lambda},\widehat{\theta})$ under expected Poisson models, since a Poisson distribution has the property of mean=variance. We choose Chi-square test statistics as distance for measuring deviation from expected models due to two reasons: 1). The derivation of joint likelihood is very time consuming, and thus the corresponding clustering algorithm is very slow and impractical for large datasets; 2) Simulation study shows that the performance of clustering algorithm using Chi-square statistics is almost as good as using joint likelihood function directly.

**PK Clustering Algorithm**

Based on the probability model described above, and *K*-means clustering method, we developed the *PK* clustering algorithm. *K*-means cluster algorithm (Hartigan, 1975) generates good clusters by specifying a desired number of clusters, say, *K*, and then assigns each object to one of *K* clusters so as to minimize a measure of dispersion within the clusters. In this work, we modified *K*-means clustering algorithm by using the Chi-square statistics as similarity measurements instead of using the Pearson correlation or Euclidean distance or other similarity measurements. The modified algorithm is called the *PK* clustering algorithm.

A brief description of the *PK* algorithm:

1. All SAGE tags are assigned at random to *K* sets. Estimate the total count $\widehat{\theta_{j}}$ of each tag by (2).
2. Set cluster centers $\widehat{\lambda_{k}^{(0)}}$by (2). $\widehat{\lambda_{k}^{(0)}}\widehat{\theta_{j}}$ then represents the expected expression pattern of the tag *j* in cluster *k*. Current iteration *i*=0.
3. In the *i*th iteration, assign each tag *j* to the cluster with the nearest expected expression pattern. We use Chi-squared statistics to measure the distance between the observed expression pattern of tag *j* (***Y****_j_*) and the expected expression pattern of tag *j* in cluster *k* ($\widehat{\lambda_{k}^{(i)}}\widehat{\theta_{j}}$):$\sum_{t} \frac{(Y_{j}(t)-\widehat{\lambda_{k}^{(i)}}(t)\widehat{\theta_{j}})^{2}}{\widehat{\lambda_{k}^{(i)}}(t)\widehat{\theta_{j}}}$
4. Set new cluster centers$\widehat{\lambda_{k}^{(i+1)}}$.
5. Go to step 3 until convergence.

In total, if *c(j)* denotes the cluster number that tag *j* is assigned to, the *PK-*clustering algorithm is to minimize the within-cluster dispersion:

$\sum_{j} \sum_{t} \frac{(Y_{j}(t)-\widehat{\lambda_{c(j)}}(t)\widehat{\theta_{j}})^{2}}{\widehat{\lambda_{c(j)}}(t)\widehat{\theta_{j}}}$.

**Implementation**

The algorithm is implemented in both C++ and Java. The routines for the EM algorithm for reassigning cluster members are from the work by Michiel de Hoon et. al. in the Human Genome Center at the University of Tokyo. The algorithm here described is available from http://genome.dfci.harvard.edu/sager.

Reference:

Hartigan, J (1975) Clustering Algorithms. John Wiley and Sons.
